# AI-Based Vehicle State Estimation Using Multi-Sensor Perception and Real-World Data

**DOI:** 10.3390/s25144253

**Published:** 2025-07-08

**Authors:** Julian Ruggaber, Daniel Pölzleitner, Jonathan Brembeck

**Affiliations:** German Aerospace Center (DLR), Institute of Vehicle Concepts, Vehicle System Dynamics and Control, 82234 Weßling, Germany

**Keywords:** vehicle dynamics state estimation, AI-based vehicle state estimation, perception data for state estimation, camera, lidar, recurrent neural network, computer vision

## Abstract

With the rise of vehicle automation, accurate estimation of driving dynamics has become crucial for ensuring safe and efficient operation. Vehicle dynamics control systems rely on these estimates to provide necessary control variables for stabilizing vehicles in various scenarios. Traditional model-based methods use wheel-related measurements, such as steering angle or wheel speed, as inputs. However, under low-traction conditions, e.g., on icy surfaces, these measurements often fail to deliver trustworthy information about the vehicle states. In such critical situations, precise estimation is essential for effective system intervention. This work introduces an AI-based approach that leverages perception sensor data, specifically camera images and lidar point clouds. By using relative kinematic relationships, it bypasses the complexities of vehicle and tire dynamics and enables robust estimation across all scenarios. Optical and scene flow are extracted from the sensor data and processed by a recurrent neural network to infer vehicle states. The proposed method is vehicle-agnostic, allowing trained models to be deployed across different platforms without additional calibration. Experimental results based on real-world data demonstrate that the AI-based estimator presented in this work achieves accurate and robust results under various conditions. Particularly in low-friction scenarios, it significantly outperforms conventional model-based approaches.

## 1. Introduction

State estimators are essential for modern vehicle technology, as they reconstruct states that are difficult to measure on the basis of available sensor measurements using mathematical models. They replace expensive, space-intensive hardware sensors using virtual software sensors, thereby offering economic advantages such as lower costs and reduced space and maintenance requirements. In recent years, the automotive industry has seen rapid development toward ever-higher levels of automation, culminating in numerous driver assistance functions and even fully autonomous driving. This trend is only made possible by modern sensor technology and intelligent data processing. In this context, vehicle state estimation is becoming increasingly important, as it provides precise information needed for a wide range of driver assistance and vehicle control functions. Advanced Driver Assistance Systems (ADASs) or vehicle dynamics control functions, such as Electronic Stability Control (ESC), require precise information about current vehicle states in order to work safely and efficiently. State estimators are therefore a key technology for the further development of modern vehicles. This work investigates the use of state estimators for vehicle dynamics control functions and highlights the potential of perception sensors such as cameras, lidars, and radars, which have been less utilized in this application area so far but offer great opportunities.

### 1.1. Motivation and Potential of Perception Sensors for Estimating Vehicle Dynamics

The vehicle dynamic states of today’s production vehicles are estimated in most cases using model-based approaches, which can range from very simple to complex vehicle models. Simple models often use a basic representation of the vehicle and rely on pure kinematic relationships. However, these approaches are inaccurate in many regions of the vehicle state space. On the other hand, more complex models provide more precise results but require detailed vehicle models, in which the main nonlinearity is represented by the tire model. This model is a central component of this dynamic system, since it describes the contact between the vehicle and the road surface. Its influence on the estimation of the vehicle states is substantial, which is why it has been the subject of research for decades. Among the best-known models is the Magic Formula by Pacejka [1], with Version 6.1 including over 200 parameters. These models often cannot be interpreted physically, which is why the model is referred to as “magic”. Alternatively, the tire model TMeasy [2] offers a better interpretability of the parameters, but also requires more than 50 values. Determining the required parameters for both models requires complex tire measurements. Furthermore, there is still uncertainty due to environmental influences such as temperature, air pressure, road conditions, or tire wear.

**Figure 1:**
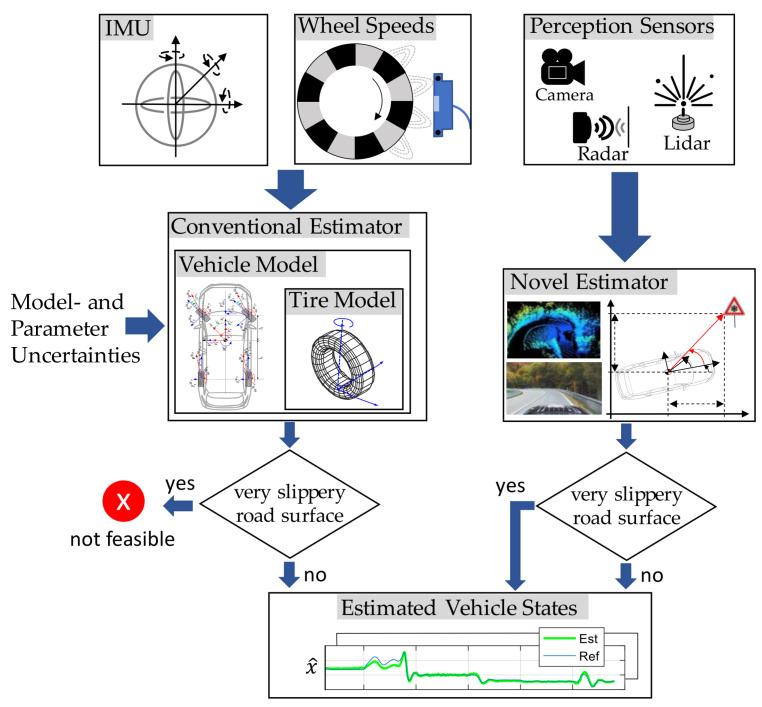
Overview of the information flow from sensors via estimators to the vehicle states: conventional approach using a vehicle model (**left**) and our proposed novel approach using perception sensors and bypassing the complex vehicle and tire model (**right**).

Many of the vehicle state estimators used today rely on these tire models and incorporate sensor information such as vehicle accelerations or angular rates from an inertial measurement unit (IMU) (see Figure 1, left part).

Recently, modern vehicles increasingly feature perception sensors such as cameras, radar, or lidar systems that can provide high-quality information about the vehicle state. These sensors make it possible to bypass the complex vehicle and tire system (see Figure 1, right part). In this case, ego-motion can be estimated, for instance, from a sequence of camera images by leveraging relative kinematic relationships, such as those observed when passing a stationary object like a traffic sign (see Figure 1).

Bypassing the tire model also allows for a robust estimate in scenarios without any traction between the tire and the road, such as when driving on ice with a friction coefficient of μ≈0. In such situations, the input variables of the vehicle model (e.g., steering wheel angle and wheel speeds) are no longer linked to the actual vehicle state, which leads to incorrect estimates in classical model-based approaches.

The novel state estimator presented in this work uses a perception model that is based solely on relative kinematic relationships and is independent of specific vehicle or environmental characteristics. The integration of modern perception sensors offers a new way to enable precise and robust vehicle dynamics state estimation—even under difficult conditions and without dependence on the tire model.

### 1.2. State of the Art

Vehicle state estimation using perception sensors is a key topic in automotive control technology. This section provides an overview of the current state of the art, dividing it into model-based and AI-based approaches. Furthermore, the work can be categorized into two main application areas:

- ADAS functions: These include systems such as lane keeping assist (LKA), traffic sign recognition, adaptive cruise control (ACC), emergency brake assist, or localization and object detection for trajectory planning
- Vehicle dynamics control (VDC): Systems such as the electronic stability program (ESC), anti-lock braking system (ABS), or other brake control systems.

In the case of ADAS functions, the localization and mapping of the ego vehicle play a key role in the state estimation. A well-established state-of-the-art method in this area is simultaneous localization and mapping (SLAM), which is available in a wide variety of modifications [3].

The bandwidth required by vehicle dynamics control systems is significantly higher than that of ADAS functions, since they must react to instabilities in real time. In contrast, ADAS functions often work predictively and have more time to process complex data from the vehicle’s surroundings [4,5]. Therefore, perception sensors are currently mainly used for ADAS functions, whereas the use of this sensor information for vehicle dynamics control is an active field of research [6]. To further explore the potential of this topic, the underlying work proposes a framework developed for applications in vehicle dynamics control estimation. The approaches to ADAS functions will not, however, be discussed in detail in the following.

#### 1.2.1. Discussion of Related Work

Table 1 shows related work on the use of perception sensors in vehicle state estimation, categorized into the methods and application areas mentioned above. The table is intended to provide a representative overview of the state of the art. Due to the multitude of different approaches and the wide range of variations, no claim is made to completeness.

The work of [7] represents a model-based approach to state estimation for VDC. The authors estimate the vehicle sideslip angle based on camera images. A vision model is used to integrate information about the lane markings into a Kalman filter. Incorporating image data improved the accuracy of the Kalman filter by almost 30 percent using a single-track model. The process was able to be operated in real time with a sampling time of 1 ms on an embedded computer.

The authors of [8] deal with the roll angle estimation for a motorcycle, which is an important control variable for the stability control system. For this purpose, the orientation of the camera images is used as a gradient, integrated in an extended Kalman filter. It was shown that a robust estimation of the roll angle was possible with an average precision of two degrees.

A framework called RAVE (RAdar Velocity Estimator) was developed in [9] to estimate the ego vehicle velocities. It is based on 3D radar measurements using a new filter method for outlier detection. Compared to other radar-based estimation methods, it was shown that outliers can be effectively detected and the estimates can be significantly improved. The framework is offered as an open-source software package and provides a Robot Operating System (ROS) interface.

In the field of AI-based approaches to vehicle state estimation, the authors of [10] presented a framework that estimates ego vehicle velocities based on camera images. In addition, the orientation, relative positions, and velocities of surrounding road users are also estimated. Using several deep neural networks, the optical flow, depth, and bounding boxes of other road users are determined using the camera images. The framework was tested with real-world measurement data, allowing speeds to be estimated with a root mean squared error (RMSE) < 0.7 m/s.

**Table 1:** Overview of related work on vehicle state estimation based on perception sensor information.

	Approach	
Application Area	Model-Based	AI-Based
**Advanced Driver** **Assistance Systems (ADAS)**	ACC-related estimates based on radar, lidar, and camera data, and Kalman filter [11] LKA-related estimates based on camera data and a multirate Kalman filter [12] Overview of localization and mapping methods, e.g., for trajectory planning [3] SLAM (Visual, lidar-based, Multi-sensor, RGB-D) LOAM Visual Odometry	Vehicle’s ego-position estimation based on Radar measurements for object detection for emergency brake assist [13] Lidar measurements using deep neural networks [14] ACC target selection based on camera and radar measurements [15] Lane Estimation with a single radar sensor using a deep learning network [16]
	….	…
**Vehicle** **Dynamics Control Systems** **(VDC)**	Via camera Estimation of the roll angle of a motorcycle from camera images by integrating the gradient of the images in a Kalman filter [8] Estimation of the vehicle’s sideslip angle via camera images of road markings and integration into a Kalman filter [7] Via radar Estimation of vehicle ego-velocities based on 3D radar measurements using a new filter method for outlier detection [9]	Via camera Estimation of ego vehicle velocity and states of surrounding traffic participants from camera images using deep neural networks [10] Via radar Estimation of ego vehicle velocities based on two 4D radar sensors using convolutional neural networks (CNN) [17] Via camera, lidar, and radar Estimation of the vehicle’s sideslip angle from camera, lidar, and radar sensor data using CNNs and recurrent neural networks (RNN) [18]

In [17], a framework is presented, which uses the point clouds from two 4D radar sensors to extract features with a convolutional neural network. These features are then used to estimate the ego vehicle velocity and vehicle rotation rates using an encoder followed by a regressor. The framework was trained and evaluated using a publicly available reference radar dataset, achieving satisfactory results.

The authors in [18] propose a framework that estimates the vehicle’s sideslip angle based on camera images, radar, and lidar point clouds. After a dimensionality reduction in the perception sensor data, a pre-trained convolutional neural network (CNN) is used to calculate optical flows. These ones are then fused with all available IMU data using an RNN. This AI estimator shows similar performance to an Extended Kalman Filter (EKF) when compared with experimental data.

In addition to the work mentioned, there are also mixed approaches that cannot be clearly assigned to any quadrant of Table 1. These include, for example, hybrid methods that use a kinematic model with AI-processed perception sensor information [19], or the combination of AI-processed camera, lidar, and radar data with a Kalman filter to estimate the ego position of a vehicle [20].

#### 1.2.2. Positioning of the Present Work

VDC requires the corresponding state estimators to be able to respond dynamically, acting in high-dynamic closed-loop control. The processing of perception sensor data must be completed in a range of milliseconds. When it comes to real-time processing of this complex data or a mix of it (image data and point clouds), AI-based approaches are becoming increasingly important thanks to their real-time capability [21]. These are being developed at a rapid pace and are already state-of-the-art in many areas of computer vision [22]. Therefore, AI-based methods were chosen for the framework presented in this work.

In this work, both camera images and point cloud data from a lidar sensor will be used to estimate the ego vehicle motion. The vehicle’s sideslip angle and vehicle velocity represent key state variables and are estimated using AI techniques for vehicle dynamics control systems. Our proposed method appears similar to the framework presented in [18] (see Table 1 quadrant no. 4). However, there are fundamental differences in the way the perception sensor data is considered: our approach uses the full 3D point cloud data and therefore does not perform any 2D dimensional reduction, which would result in the loss of valuable information. Moreover, instead of dense optical flow used in [18], our approach makes use of sparse optical flow. This results in a significantly more efficient neural network structure and has led to considerably better generalizability and estimation results in our experiments.

### 1.3. Contribution of This Work

As outlined and discussed in the preceding sections, this work introduces several novel contributions across different aspects. These can be summarized as follows:

- The perception sensors are used for high-dynamic functions of vehicle dynamics control systems instead of less dynamic top-level ADAS applications
- By using the tire-independent perception sensor information, two major improvements arise for a vehicle dynamics state estimator:
- Ease of application and transferability of the estimator to vehicle platforms of any kind. Trained AI-based vehicle state estimators using perception sensors can be transferred to any other vehicle without the need for adaptations.
- Robustification of vehicle state estimation. Estimation is also possible in driving scenarios, in which there is no longer any traction between the tires and the road (e.g., when driving on an icy surface).
- The state variables that are estimated are the vehicle sideslip angle as a safety-critical variable, as well as the vehicle velocity, which is one of the most essential vehicle states representing a basic variable for calculating a number of other VDC quantities.
- The fundamental kinematic relationships between the vehicle states and the perception sensor data are derived to gain a comprehensive understanding of the interactions.
- The performance of the developed toolchain is analyzed using real-world measurement data from test drives and compared with proven model-based methods, such as an Unscented Kalman Filter (UKF).

Overall, this work aims to enhance the accuracy, robustness, and transferability of vehicle state estimation, thereby addressing the practical requirements of modern vehicle dynamics control systems and enabling their effective application across a wide range of vehicle platforms and driving scenarios.

## 2. System Architecture of the AI-Based Vehicle State Estimator Utilizing Perception Sensors

**Figure 2:**
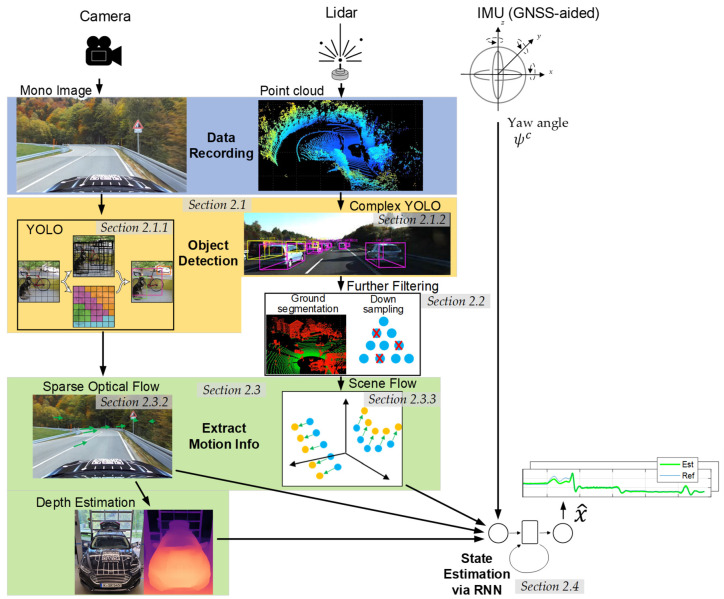
Overview of the complete tool chain representing the vehicle state estimator. Symbol images for object detection were sourced from You Only Look Once (YOLO) [23] and Complex YOLO [24], while the remaining elements were designed by the authors. The depicted toolchain is referred to as the ‘Novel Estimator’ (right-hand side) in Figure 1.

The AI-based state estimator proposed in this work uses mono images from a camera and point cloud data from a lidar sensor to perceive the vehicle environment. The tool chain could be extended to include a radar sensor. This data is processed analogously to the point cloud data of the lidar sensor. In addition to the perception sensors, the GNSS (Global Navigation Satellite System)/IMU platform is used to incorporate the vehicle yaw angle. This is necessary to separate the vehicle sideslip angle from the course angle, and is described in Section 2.3.1 in more detail. Further information on the technical specification of the sensors and their mounting and integration in the vehicle is provided in Section 3.1 and Appendix B. An overview of the complete tool chain of the state estimator is depicted in Figure 2.

This chapter presents various steps of the tool chain and briefly explains their underlying functional principles. Section 2.1 illustrates that object detection must first be carried out on the recorded perception sensor data. This is necessary since it is not possible to reconstruct the ego motion based on dynamic road users (e.g., oncoming traffic). For the detection, the well-known You Only Look Once (YOLO) framework [23] is exploited for the image data, and Complex YOLO [24] is used for the point cloud data. These algorithms detect dynamic objects and filter them out of the data. The lidar point cloud, which contains extensive information about the vehicle’s environment, requires preprocessing steps like ground segmentation and downsampling, which are discussed in Section 2.2. The next section explains the underlying theory for extracting information about the ego vehicle’s dynamics from its environment. Section 2.3.2 and Section 2.3.3 present the calculation of the optical flow from the image data and the scene flow from point cloud data, respectively. The depth estimation from the image data required to correctly incorporate the sparse optical flow in an RNN is shown in Section 2.3.2. The last section concludes how the aforementioned variables (together with the vehicle yaw angle $\psi^{c}$) are fused in a recurrent neural network (RNN) and trained to determine the vehicle sideslip angle $\beta^{c}$ and velocity $v^{c}$.

### 2.1. Detection of Dynamic Interfering Objects

**Figure 3:**
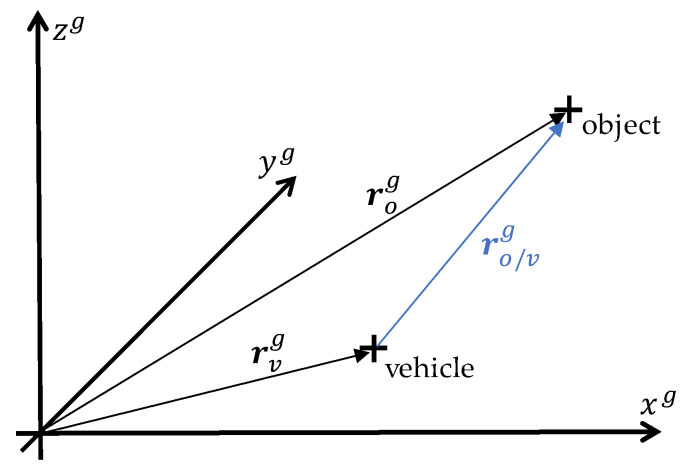
Overview of the relationship between the vehicle, the sensors installed on it, and a detected object.

The basic idea for estimating the ego-motion is based on the use of perception sensors that, mounted on the vehicle ${r_{v}^{g}(t)=\left(x_{v},y_{v},z_{v}\right)}^{g}$, move within a geodetic coordinate system. These sensors capture the surrounding environment and detect the motion relative to stationary objects, with the position $r_{o}^{g}(t)={\left(x_{o},y_{o},z_{o}\right)}^{g}$ (see Figure 3).

Since the observed relative motion of these objects is caused solely by the motion of the sensors on the vehicle, a back-calculation can be used to determine its ego-motion. Dynamic objects (e.g., other vehicles or pedestrians) move independently of the observer. These objects cannot be used to estimate ego-motion because their motion is unknown. Therefore, they must be filtered out of the image and point cloud data.

**Statement:** The movement of dynamic objects provides no information about the ego motion.

**Proof.**  Given the position of the moving vehicle $r_{v}^{g}(t)={\left(x_{v}\left(t\right),y_{v}\left(t\right),z_{v}\left(t\right)\right)}^{g}$ and the position of an object point $r_{o}^{g}(t)={\left(x_{o}(t),y_{o}(t),z_{o}(t)\right)}^{g}$, the relative position of the object with respect to the vehicle is:

(1) $$r_{o/v}^{g}(t)=r_{o}^{g}\left(t\right)-r_{v}^{g}\left(t\right).$$

The relative velocity is given by the time differentiation of Equation (1):

(2) $${\dot{r}}_{o/v}^{g}(t)={\dot{r}}_{o}^{g}\left(t\right)-{\dot{r}}_{v}^{g}\left(t\right).$$

A perception sensor, such as a stereo camera or a lidar, is capable of measuring the relative position $r_{o/v}^{g}(t)$ and relative velocity ${\dot{r}}_{o/v}^{g}(t)$ of the object w.r.t. to the vehicle. In order to estimate the vehicle’s ego motion ${\dot{r}}_{v}^{g}\left(t\right)$, Equation (2) can be resolved to:

(3) $${\dot{r}}_{v}^{g}\left(t\right)={\dot{r}}_{o}^{g}\left(t\right)-{\dot{r}}_{o/v}^{g}(t).$$

Hence, Equation (3) is underdetermined without any information about the object’s motion ${\dot{r}}_{o}^{g}\left(t\right)$. Only if the object is stationary, i.e., ${\dot{r}}_{o}^{g}(t)=0$, can the vehicle’s ego motion be uniquely calculated as:

(4) $${\dot{r}}_{v}^{g}\left(t\right)=-{\dot{r}}_{o/v}^{g}(t).$$

 □

#### 2.1.1. Object Detection in Image Data via YOLO

There are a wide variety of methods for detecting objects in images. Classic approaches such as the *Deformable Parts Model* or *Histogram of Oriented Gradients* are increasingly being outperformed by AI-based methods [25], which often involve CNNs. There are two-stage techniques that perform localization and classification. They generally achieve a high level of accuracy but require high computational effort. In contrast, one-stage methods are less accurate but faster and more efficient, which makes them more suitable for real-time applications. The probably best-known method for one-stage methods is YOLO (You Only Look Once) [23], which is widely used in both research and industry. YOLO was introduced in 2015 and has been continuously developed since then, steadily increasing its popularity and performance [26]. The algorithm divides an image into a grid of cells. Object detection is carried out for each cell, whereby bounding boxes and class probabilities are determined in each case. In the final step, overlapping bounding boxes are removed. YOLO detects and classifies input images in a single pass through the CNN, making it extremely fast.

**Figure 4:**
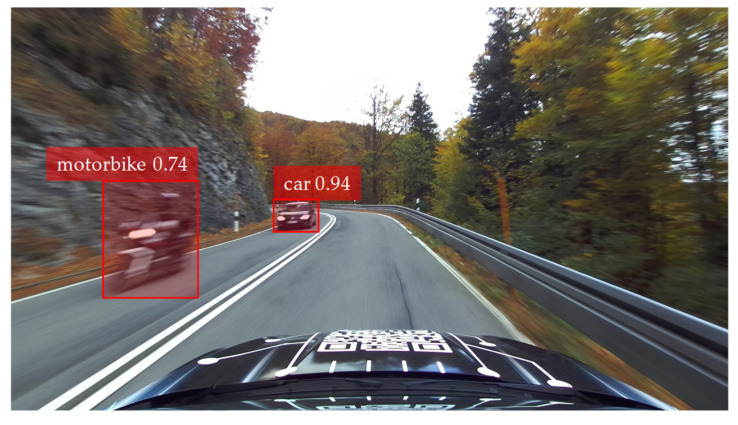
Object detection via YOLOv4. The camera is mounted directly above the windshield and captures the area in front of the vehicle.

For the vehicle state estimation framework in this work, a YOLOv4 implementation in TensorFlow is used [27]. The CNN employed was trained using the well-known *Microsoft Common Objects in Context* (COCO) dataset [28]. Thus, dynamic objects such as people, cyclists, cars, and trucks, which are relevant for this work, can be identified. Figure 4 shows an example of an image evaluated in this way, which was taken with a camera mounted above the windshield of our research vehicle during a test drive.

The bounding boxes for the dynamic objects identified by YOLO that meet the confidence threshold of ≥0.5 are removed from the images because these objects are interfering with the ego-motion estimation (see Section 2.1).

A given image can be described by a matrix $I\in R^{w\times l}$, where w and l describe the width and length, respectively. The $n_{obj}$ dynamic objects identified by YOLO are described by a set of masks $M^{1},M^{2},\ldots ,M^{n_{obj}}$, where each mask $M^{i}\in R^{w\times l}$ is a binary matrix with the respective values:

(5) $$M_{w,l}^{i}=\left\{\begin{array}{c} 1\ldots dynamic\;object\;detected,\\ 0\ldots otherwise. \end{array}\right.$$

The overall mask for image I is the pixel-by-pixel logical OR of the individual masks:

(6) $$M_{total}={⋁}_{i=1}^{n_{obj}}M^{i}.$$

The $n_{obj}$ dynamic objects are masked out of the image I by setting their RGB pixel values to 0 (black). By applying the pixel-wise product (Hadamard product), it follows that

(7) $$I_{masked}=I⊙\left(1-M_{total}\right),$$

where **1** denotes a matrix of ones with the same dimensions as $M_{total}$.

#### 2.1.2. Object Detection in Point Clouds via Complex YOLO

**Figure 5:**
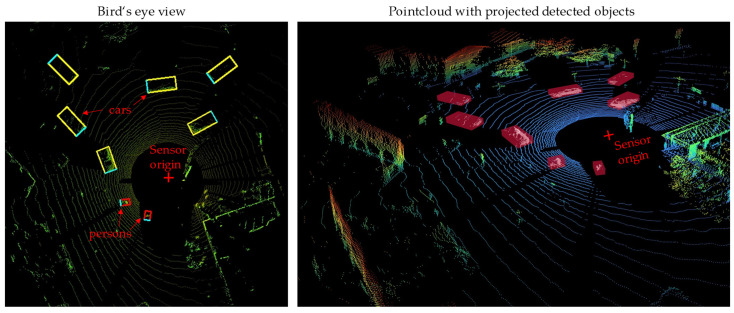
Applying Complex-YOLO to a lidar frame during an urban test drive. **Left**: bird’s eye view image transformed from the point cloud, which is the basis for the dynamic object detection. **Right**: original point cloud and the projected bounding boxes of the detected objects.

There are a variety of approaches available for object detection in point cloud data [29]. For the reasons discussed in Section 2.1, which primarily concern real-time capability and efficiency, an AI-based approach is used for the point clouds in the vehicle state estimation framework. To this end, an extension of the YOLO algorithm capable of handling 3D data is used for image data. With the help of this extension called Complex-YOLO [24], objects can be identified in a point cloud. In this work, a Pytorch implementation of Complex-YOLOv4 is used [30]. The algorithm transforms a 3D point cloud into a 2D bird’s-eye view RGB image. This 2D image is processed by a modified YOLOv4 architecture, which estimates the orientation of the recognized objects using an Euler region proposal network. The recognized 2D bounding boxes are then projected into a 3D space, where the position, size, and angle estimation are carried out in real time [24]. Figure 5 shows the object detection of Complex-YOLO for a point cloud during a test drive we performed. The point cloud consists of a set of 65,536 points (the interested reader is referred to Appendix B for more information about the technical specifications).

The detected dynamic objects must be removed from the point cloud because, analogously to the camera images, they represent interference with the ego-motion estimation. The three-dimensional point cloud P considered at a fixed frame is defined as a set of points:

(8) $$P=\left\{p_{1},p_{2},\ldots ,p_{65536}\right\},\;p_{i}\in R^{3}$$

with the individual points $p_{i}=\left(x_{i},y_{i},z_{i}\right)$, which are described by their spatial coordinates. The recognized dynamic objects are defined as clusters of points $O_{dyn,j}\subset P$, for $j=1,\ldots ,n_{obj}$, which can be described for a frame as:

(9) $$O_{dyn,total}={⋃}_{j=1}^{n_{obj}}O_{dyn,j}.$$

The point cloud cleared of dynamic objects can then be expressed as:

(10) $$P_{non\;dyn}=P\setminus O_{dyn,total}.$$

### 2.2. Preprocessing the Lidar Point Cloud

Prior to determining a motion from the point clouds using scene flow (see next section), further preprocessing is necessary. This includes ground segmentation to eliminate the ground for the scene flow calculation. In addition, a region of interest (ROI) can be defined by an advantageous preselection. Finally, downsampling helps to considerably reduce the number of relevant points.

#### 2.2.1. Ground Segmentation

The scene flow resulting from the vehicle motion is calculated based on the difference of the points between two frames (see next section). Since the ground generally has a low texture and only limited visual features, calculating the scene flow based on these points is very difficult. This applies in particular to homogeneous surfaces such as asphalt [31]. For this reason, the points that belong to the ground should not be used for further calculations.

The point cloud without dynamic objects from Equation (10) $P_{non\;dyn}\subseteq R^{3}$ is given, with the individual points $p_{i}=\left(x_{i},y_{i},z_{i}\right)$. The challenge for ground segmentation arises from the fact that the ground is not a flat surface. Instead, the ground is defined as a continuous surface:

(11) $$G=\left\{\left(x_{g},y_{g},z_{g}\right)\in R^{3}|z-f\left(x,y\right)=0\right\},$$

where the function $f\left(x,y\right)$ describes the relative height of the ground. The set of points that do not belong to the ground is then:

(12) $$P_{non\;ground}=\left\{\left(x,y,z\right)\in P_{non\;dyn}|z-f\left(x,y\right)>0\right\},$$

and the points that represent the ground:

(13) $$P_{ground}=\left\{\left(x,y,z\right)\in P_{non\;dyn}|z-f\left(x,y\right)=0\right\}.$$

For the relationships described above, the following properties apply:

(14) $$P_{non\;ground}\;\cap P_{ground}=\emptyset ,\;P_{non\;dyn}=P_{non\;ground}\cup P_{ground}.$$

**Figure 6:**
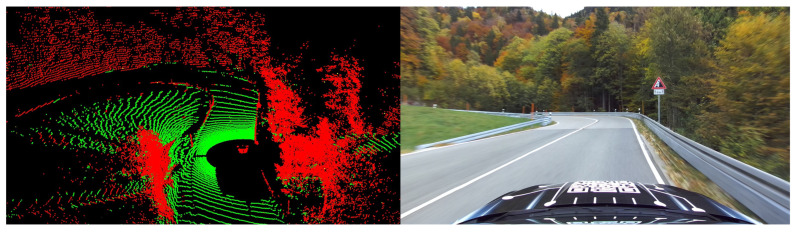
**Left**: The ground segmentation of a lidar frame for a serpentine drive. Green dots correspond to the ground, red dots to non-ground. **Right**: Image from the camera for the reader’s better understanding of the scenario.

The authors of [32] presented an algorithm called *Patchwork++* that solves the ground segmentation problem efficiently and with low computational effort. To this end, the data is divided into concentric zones with a separate ground plane fitted for each zone, thus taking local variations into account. Figure 6 shows a point cloud frame that was recorded during one of our test drives and to which ground segmentation was applied.

The open-source Python implementation of *Patchwork++* is used for the vehicle state estimation framework in this work. The ground segmentation algorithm has proven its efficiency in this challenging scenario with a serpentine road. First, it is capable of identifying ground points lying at different relative heights during the uphill drive. Second, the algorithm can reliably segment together different ground surfaces, such as road and meadow. As discussed at the beginning of the section, only the non-ground points $P_{non\;ground}$ are used for further processing.

#### 2.2.2. Considering a Region of Interest

Not all remaining points of the point cloud should be used to extract motion information. The scene flow calculation carried out in Section 2.3.3. can be significantly improved by a careful point pre-selection. This reduction in the point cloud can be performed by defining a region of interest (ROI). The following aspects should be considered during its selection:

- The lidar sensor used has a range of up to 200 m. However, points at large distances ≳50 m are associated with a high degree of uncertainty or noise and should not be used.
- Due to the structural characteristics of a road, the longitudinal environment should be considered at a longer distance than the lateral environment.
- The maximum height of the points considered should be limited in order to avoid reflections, for example, in moving treetops.

**Figure 7:**
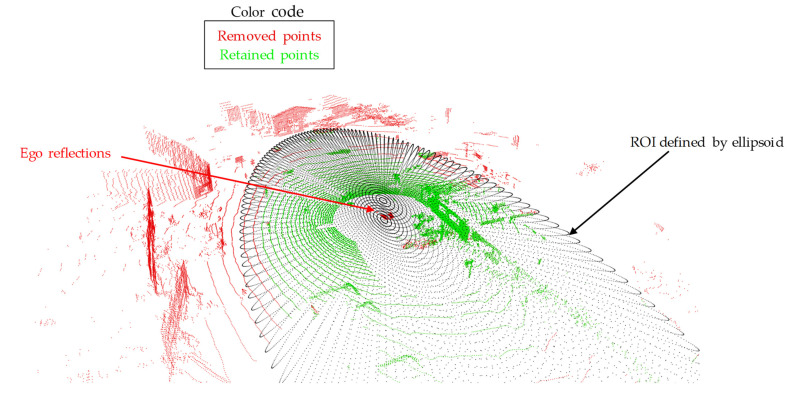
The region of interest for the lidar point cloud is implemented using an ellipsoid. Red points are removed, green points are kept. The ground is shown for visualization purposes, but is actually removed beforehand by ground segmentation.

These considerations are implemented by an ROI, which can be described by an ellipsoid (see Figure 7).

The center of the ellipsoid is located at the origin of the lidar sensor and has the three semi-axes $x_{max},\;y_{max}$, and $z_{max}$, which define the maximum longitudinal, lateral, and vertical expansion of the ROI. All points that fulfill the ellipsoid equation lie within the ROI:

(15) $$P_{ROI}=\left\{\left(x,y,z\right)\in P_{non\;ground}|\frac{x^{2}}{x_{max}^{2}}+\frac{y^{2}}{y_{max}^{2}}+\frac{z^{2}}{z_{max}^{2}}\leq 1\right\}.$$

All other points lie outside this area and are not used for further processing of the point cloud. In addition, ego reflections are filtered out. These occur, for example, since parts of the vehicle’s own roof are illuminated by the lidar.

#### 2.2.3. Downsampling

The original point cloud captured by the lidar contains over 65,000 points. Following the filtering of dynamic objects (Section 2.1.2), the ground segmentation (Section 2.2.1), and the consideration of the ROI (Section 2.2.2), the point cloud still consists of more points (in most of the cases we examined ≈10,000 points) than are later required for extracting the motion. This number of points contains multiple redundancies of the vehicle’s ego motion, cf. Equations (26) and (27). To reduce this redundancy and also the complexity, the point cloud is therefore downsampled. For this purpose, an index-based selection of the points is performed, in which the sequence of points in $P_{ROI}=\left\{p_{1},p_{2},\ldots ,p_{n}\right\}$ is reduced evenly. This down-sampling results in a subset $P_{final}\subset P_{ROI}$, which contains only every k-th triple $p_{i}=\left(x_{i},y_{i},z_{i}\right)$ from the point cloud $P_{ROI}$:

(16) $$P_{final}=\left\{p_{i}\in P_{ROI}|i\;mod\;k=1\right\}.$$

The order of $p_{i}$ is determined by the sequential, line-by-line scanning process of the lidar sensor. The lines, stacked vertically, collectively form the point cloud representing the entire environment. In our investigations, k=4 has proven to be a suitable reduction factor.

### 2.3. Motion Extraction from Perception Sensors

In this section, the basic mathematical relationships are derived that describe how information about the vehicle motion can be extracted from the perception sensors. Therefore, the relative kinematic relationships between the vehicle’s environment and its ego motion are first described. Based on these findings, it is explained how the optical flow of camera images (Section 2.3.2) and the scene flow of lidar point clouds (Section 2.3.3) can be used for this purpose. The subsequent recurrent neural network (RNN) fuses the extracted motion information together with the IMU/GNSS system (Section 2.4) and represents the actual state estimator.

Understanding the relationships that the RNN is supposed to learn is the basic requirement for meaningful (structural) network design and for avoiding a complete black box view.

#### 2.3.1. Relative Kinematics of the Vehicle Environment and Its Dynamics

**Figure 8:**
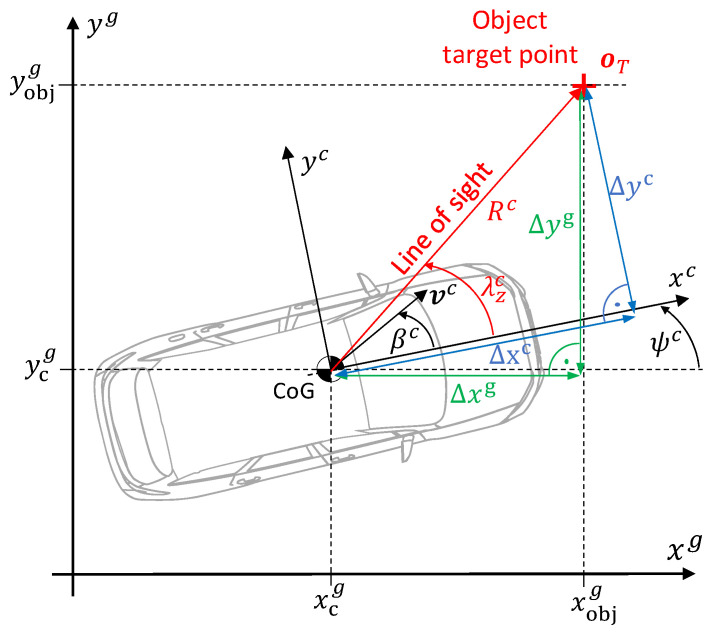
Overview of the relative kinematic relationships between the vehicle environment, i.e., the object, and the vehicle itself.

Two of the most important quantities for describing driving dynamics are the vehicle sideslip angle $\beta^{c}$ and the vehicle velocity $v^{c}$, which are defined in a vehicle-fixed coordinate system ${\left(\cdot \right)}^{c}$ (see Figure 8). The estimation of these quantities is the goal of the approach presented in this paper.

The vehicle velocity is the magnitude of the velocity vector located at the center of gravity (CoG):

(17) $$v^{c}=\sqrt{{v_{x}^{c}}^{2}+{v_{y}^{c}}^{2},}$$

whereby the components $v_{x}^{c}$ and $v_{y}^{c}$ represent the longitudinal and lateral velocities. The vehicle sideslip angle $\beta^{c}$ describes the angle between the longitudinal vehicle axis $x^{c}$ and the velocity vector $v^{c}$, that is, the angle between the longitudinal and lateral velocity:

(18) $$\beta^{c}=\tan^{-1}\left(\frac{v_{y}^{c}}{v_{x}^{c}}\right).$$

The longitudinal vehicle axis $x^{c}$ is rotated by the yaw angle $\psi^{c}$ in the geodetic coordinate system (see Figure 8).

An object surrounding the vehicle is described by the Target Point $o_{T}$. The direct connection between the vehicle’s CoG and the object is called the line of sight. This line is oriented around the so-called look angle $\lambda_{z}^{c}$ to the vehicle longitudinal axis. The length of the line of sight is the distance from the object to the vehicle $R^{c}$. This distance can be divided into its longitudinal and lateral components in the vehicle-fixed coordinate system $\Delta x^{c}$ and $\Delta y^{c}$. Here, we assume a planar perspective, disregarding any contributions from the z-component.

**Note:** This representation assumes that the sensor coordinate system is identical to the vehicle coordinate system. In practice, the sensors are not located at the position like the CoG, which is why the motion extracted from them must be transformed into the CoG using appropriate algorithms.

The following relationships are described in the geodetic coordinate system, as this inertial frame offers significant advantages for analysis. In particular, its non-rotating nature simplifies the computation of time derivatives and enhances the clarity and interpretability of the principles. Accordingly, the longitudinal and lateral distances, $\Delta x^{c}$ and $\Delta y^{c}$ are transformed into the geodetic coordinate system using the direction cosine matrix $C_{c}^{g}$ and the vehicle yaw angle $\psi^{c}$:

(19) $$\left[\begin{array}{c} \Delta x^{g}\\ \Delta y^{g} \end{array}\right]=\underset{=C_{c}^{g}}{\underbrace{\left[\begin{array}{cc} \cos\left(\psi^{c}\right) & -\sin\left(\psi^{c}\right)\\ \sin\left(\psi^{c}\right) & \cos\left(\psi^{c}\right) \end{array}\right]}}\cdot \left[\begin{array}{c} \Delta x^{c}\\ \Delta y^{c} \end{array}\right].$$

Based on the geometric relationships, the following applies to the look angle:

(20) $$\lambda_{z}^{c}=\tan^{-1}\left(\frac{\Delta y^{g}}{\Delta x^{g}}\right)-\psi^{c}.$$

For the look angle rate, we obtain (the derivation is provided in Appendix A):

(21) $${\dot{\lambda }}_{z}^{c}=\frac{v^{c}}{R^{c}}\sin\left(\lambda_{z}^{c}-\beta^{c}\right)-\dot{\psi^{c}}.$$

Instead of the geometric derivation for ${\dot{\lambda }}_{z}^{c}$, an algebraic relation by differentiation of Equation (20) leads to:

(22) $${\dot{\lambda }}_{z}^{c}=\frac{\Delta x^{g}\dot{\Delta y^{g}}-\Delta y^{g}\dot{\Delta x^{g}}}{\Delta {x^{g}}^{2}+\Delta {y^{g}}^{2}}-\dot{\psi^{c}}.$$

For the distance between the vehicle and the object, the following applies:

(23) $$R^{c}=\sqrt{\Delta {x^{g}}^{2}+\Delta {y^{g}}^{2}}.$$

The approach velocity can be expressed geometrically as (the derivation is provided in Appendix A)

(24) $$\dot{R^{c}}=-v^{c}\cdot \cos\left(\lambda_{z}^{c}-\beta^{c}\right),$$

or algebraically by differentiating Equation (23) with respect to time

(25) $${\dot{R}}^{c}=\frac{\Delta x^{g}\dot{\Delta x^{g}}+\Delta y^{g}\dot{\Delta y^{g}}}{\sqrt{\Delta {x^{g}}^{2}+\Delta {y^{g}}^{2}}}.$$

The geometric and algebraic Expressions (21)–(25) can now be transformed into the following two relationships using the look angle from Equation (20):

(26) $$-v^{c}\cos\left(\tan^{-1}\left(\frac{\Delta y^{g}}{\Delta x^{g}}\right)-\psi^{c}-\beta^{c}\right)=\frac{\Delta x^{g}\dot{\Delta x^{g}}+\Delta y^{g}\dot{\Delta y^{g}}}{R^{c}},$$

(27) $$\frac{v^{c}}{R^{c}}\sin\left(\tan^{-1}\left(\frac{\Delta y^{g}}{\Delta x^{g}}\right)-\psi^{c}-\beta^{c}\right)=\frac{\Delta x^{g}\dot{\Delta y^{g}}-\Delta y^{g}\dot{\Delta x^{g}}}{\Delta {x^{g}}^{2}+\Delta {y^{g}}^{2}}.$$

These two equations contain, in addition to the states $v^{c}$ and $\beta^{c}$, only quantities that can be measured with the help of perception sensors (for the exact calculation steps, see Section 2.3.2 and Section 2.3.3). The required quantities are summarized for each sensor type in Table 2.

In addition, the yaw angle $\psi^{c}$ from the vehicle IMU (GNSS-aided) is required.

There are two Equations (26) and (27) and two unknowns (the vehicle states $v^{c}$ and $\beta^{c}$ that are being estimated). These two equations apply to each individual object point. This means that for $n_{obj}>2$ object points detected by the perception sensors, there is an over-determined, nonlinear system of equations of dimension $2\times n_{obj}$. In principle, this system of equations could be solved using classical methods such as nonlinear least squares.

However, there are good reasons to learn the relative kinematic relationship between Equations (26) and (27) using a data-based approach, i.e., by using an artificial neural network. In fact, the variables required for the equations in Table 2 are, with the exception of the lidar distance and lidar position, only estimated quantities (see Section 2.3.2 and Section 2.3.3). As a result, the variables contain uncertainties and noise, which should not be underestimated depending on the scenario. By definition, environmental conditions and associated uncertainties heavily influence the measurements of the perception sensors (exposure conditions like rain, snow, etc.). A learning-based approach demonstrates robust capabilities in handling noisy and uncertain data, enabling the reliable recognition of patterns and relationships within disturbed datasets and facilitating precise predictions and solutions [33]. Therefore, in our work, data from the perception sensor is used in a data-driven approach, specifically a recurrent neural network (RNN), to learn the relationships between the sensor readings and the desired vehicle states (see Section 2.4).

The analysis of the relative kinematic relationships between the vehicle sideslip angle $\beta^{c}$ and the vehicle velocity $v^{c}$ are represented in Equations (26) and (27), as well as in the quantities required from the perception sensors (Table 2). Based on these findings, the choice of the input variables of the RNN can be determined. The calculation of these quantities, which need to be extracted or estimated from the perception sensors, is described in the following two sections.

#### 2.3.2. Motion Extraction from Camera Image Using Optical Flow

The camera records mono images of the area in front of the vehicle. The required quantities (listed in Table 2) must now be calculated from these mono images in order to obtain the information about the vehicle sideslip angle and the vehicle velocity.

**Calculating Sparse Optical Flow**

The relative positional change from the object to the vehicle in the image $\dot{\Delta x^{c}},\dot{\Delta y^{c}}$ can be represented by the optical flow. It describes the motion of image points $\left(x^{img},y^{img}\right)$ in an image sequence, which results from the relative motion between the camera and the scene. The image coordinates $\left(x^{img},y^{img}\right)$ are discrete pixel positions within a 2D image matrix. These image coordinates are related to the 3D world points $\left(\Delta x^{c},\Delta y^{c},\Delta z^{c}\right)$ by the so-called camera projection matrix [34]. This transformation includes both the intrinsic camera properties (focal length and optical center) and extrinsic parameters (position and orientation of the camera with respect to the world coordinate system). Assuming that a pixel remains constant in brightness or intensity I during a time step Δt and a motion of ${\Delta x}^{img},{\Delta y}^{img}$, the following equation [35] results:

(28) $$I\left(x^{img}+{\Delta x}^{img},y^{img}+{\Delta y}^{img},t+\Delta t\right)=I\left(x^{img},y^{img},t\right).$$

For small movements or small time steps, Equation (28) can be approximated using a first-order Taylor expansion [35]:

(29) $$\frac{\partial I}{\partial x^{img}}{\dot{x}}^{img}+\frac{\partial I}{\partial y^{img}}{\dot{y}}^{img}+\frac{\partial I}{\partial t}=0,$$

where ${\dot{x}}^{img}$ and ${\dot{y}}^{img}$ represent components of the optical flow in the longitudinal and lateral directions. Equation (29), which is referred to as the optical flow equation, contains the two unknowns ${\dot{x}}^{img}$ and ${\dot{y}}^{img}$ and is therefore underdetermined. With additional assumptions, such as smoothness of the flow field, this equation can be solved, and the optical flow can be determined [36]. For an image represented by an intensity matrix $I\left(x^{img},y^{img},t\right)$, the optical flow field can be described as a vector field:

(30) $$F_{OF}\left(x^{img},y^{img},t\right)=\left[\begin{array}{c} {\dot{x}}^{img}\left(x^{img},y^{img},t\right)\\ {\dot{y}}^{img}\left(x^{img},y^{img},t\right) \end{array}\right],$$

where each pixel $x^{img},y^{img}\in I$ is associated with an optical flow vector.

Optical flow can be categorized into two types—dense and sparse—based on the image points for which the flow is computed.

In dense optical flow, the motion vectors are determined for each pixel in the image. A widely used method for its calculation is the Farneback algorithm [37]. Dense optical flow provides a complete representation of the motion, but is relatively computationally intensive and has problems calculating homogeneous image areas (i.e., regions with little or no texture or intensity variation) [38]. In contrast, the sparse optical flow only calculates the motion vectors for selected image points. Points with interesting image features, such as corners or edges, are used here, which can be well tracked within an image sequence [39]. A common algorithm for calculating the sparse optical flow is the Lucas–Kanade method [39].

**Figure 9:**
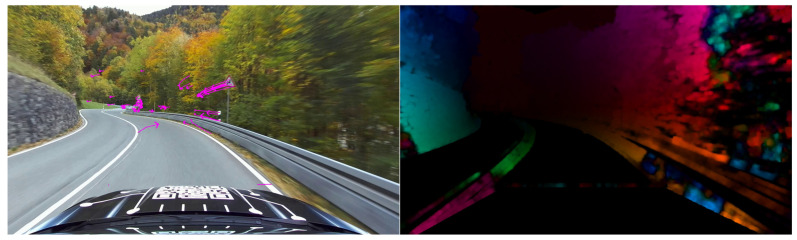
Comparison between the sparse (**left**) and the dense optical flow (**right**).

Both approaches were examined in the context of the vehicle state estimator framework presented here. Figure 9 shows the comparison between the sparse and dense optical flow. While the flow vectors are clearly visible in the sparse case, the dense variant is represented by a converted RGB image: the color represents the direction of the flow vector, the brightness indicates the length of the flow vector (the brighter a pixel, the greater its displacement).

For dense optical flow, a Full HD image resolution with 1920×1080 pixels results in a total of >4 million flow vectors (each pixel has a longitudinal and a lateral flow component). Due to the required data handling, this number entails a considerable computational effort.

Furthermore, the question arises regarding the necessity of this high number of flow vectors when applying this method to vehicle state estimation. In fact, there is an extreme redundancy, and the subsequent extraction of the vehicle states causes enormous problems. The RNN, which has fully connected layers, thus acquires an extremely complex structure and is, in many cases, no longer capable of generalization.

Our investigations have shown that flow vectors of only 20 image points are sufficient to extract the driving states.

**Note:** According to Equations (26) and (27), just two image points are theoretically sufficient to solve the underlying system of equations for the vehicle states. In practice, however, more points should be used to better account for measurement uncertainties and sensor noise.

To realize a processing chain that is as efficient as possible without a loss of performance, the sparse optical flow is used for the vehicle state estimation framework. The Lucas–Kanade method [39], which is implemented in OpenCV [40], is exploited for this purpose.

**Depth Estimation from Mono Camera Image**

As explained in Section 2.3.1, the distance from an object to a sensor $R^{c}$ is also required to reconstruct the vehicle states from the camera image. This necessity is also intuitively clear, since the optical flow of faraway objects is significantly lower than that of nearby objects and must therefore be taken into account accordingly. In the case of a mono camera image, the depth information is not available, unlike in the case of a stereo camera image, and must therefore be approximated.

To estimate the depth, we use the open-source model Monocular Depth Estimation via Scale (MiDaS) [41], which was developed by Intel Labs. MiDaS uses artificial neural networks to estimate the relative depth for each pixel from a single RGB image. The algorithm is provided in different model variants that allow a trade-off between computational effort and accuracy.

**Figure 10:**
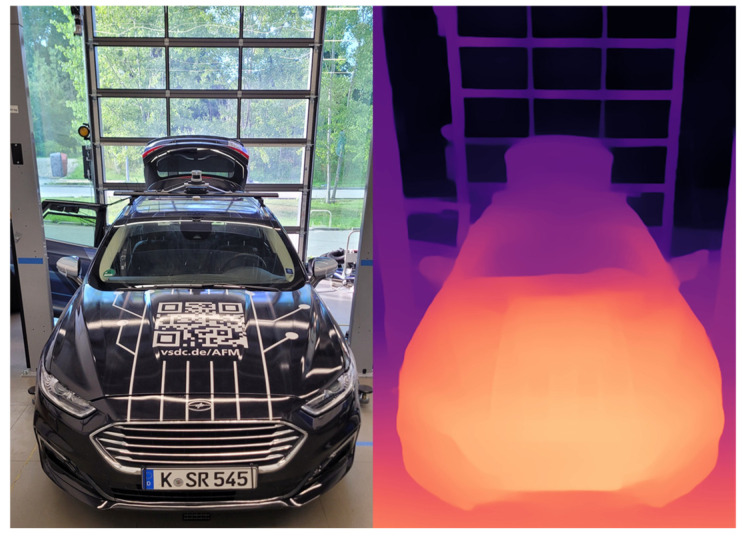
Depth image of the AFM research vehicle generated by MiDaS. Light areas represent nearby objects, dark ones represent more distant objects.

Figure 10 shows an example depth image, on which the DLR research vehicle AI For Mobility (AFM) [42] can be seen (see Section 3.1). Light areas represent close objects, whereas dark areas correspond to objects that are located further away from the camera.

MiDaS estimates the relative distances between the pixels in a scene, which means that the depth values are not absolute metric distances. This relative depth $d_{rel}$ is proportional to the actual depth and is displaced by b. With two known reference points in the image (e.g., the vehicle hood, c.f. Figure 9), the scaling factor k and b can be determined, thus enabling the calculation of the absolute depth [41]:

(31) $$R_{MiDaS}=\frac{1}{k\cdot d_{rel}+b}.$$

**Determining Relative Distances to an Object**

To reconstruct the vehicle states, the final quantities required from a camera image are the longitudinal and lateral distances from the camera to the objects $\Delta x^{c},\Delta y^{c}$ as explained in Section 2.3.1. The challenge here again lies in the fact that only a mono image is available from the camera. However, with the previously estimated depth information, it is now possible to calculate the object distances required. With the help of the intrinsic camera parameters, the position of a recorded object can be reconstructed in a three-dimensional coordinate system. The image coordinates $x^{img}$ (longitudinal) and $y^{img}$ (lateral) are related to the world coordinates $\left(\Delta x^{c},\Delta y^{c},\Delta z^{c}\right)$ by the so-called collinearity equation [43] and the relative geometry considering the distance $R_{MiDaS}^{c}$ from Equation (31):

(32) $$\Delta y^{c}=R_{MiDaS}^{c}\cdot \frac{y^{img}-c_{y}}{f},\;\Delta x^{c}=\sqrt{{R_{MiDaS}^{c}}^{2}-\Delta y^{c}},$$

where f is the focal length and $c_{y}$ is the principal point of the camera (intrinsic parameters).

It should be pointed out that the depth estimation by MiDaS is inherently uncertain, particularly for distant points or textureless surfaces. Although uncertainties influence the overall accuracy of the system, the RNN is capable of tackling this issue through the integration of potentially unreliable depth information with other motion data.

#### 2.3.3. Motion Extraction from Lidar Point Clouds Using Scene Flow

The point clouds recorded by the lidar sensor have already been preprocessed as described in Section 2.2. This includes eliminating dynamic objects using Complex YOLO, removing the ground using ground segmentation, retaining points inside the ROI, and uniform down-sampling.

For each frame at time t, this preprocessing is performed, resulting in point clouds $P_{final,t}\subseteq R^{3}$ with the respective individual points $p_{i}=\left(x_{i},y_{i},z_{i}\right)$ (see Section 2.2.3). In order to reconstruct the two vehicle states of interest, the relative kinematic information summarized in Table 2 must be extracted based on these point clouds.

The distance between the sensor and the object point i can be calculated directly using the Euclidean distance:

(33) $$R_{Lidar,i}^{c}=\sqrt{x_{i}^{2}+y_{i}^{2}+z_{i}^{2}}.$$

The longitudinal and lateral distance between the sensor and the object point i is measured directly and does not require any conversion:

(34) $$\Delta x^{c}=x_{i},\;\Delta y^{c}=y_{i}.$$

**Figure 11:**
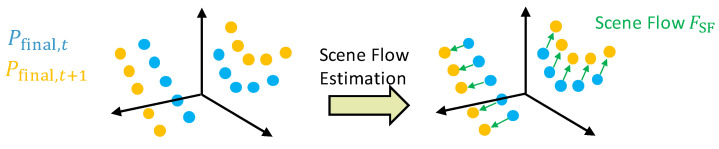
Visualization of a scene flow, which describes a 3-dimensional motion of the points between two consecutive point clouds.

The change in object position relative to the vehicle $\dot{\Delta x^{c}},\dot{\Delta y^{c}}$ is the so-called scene flow—the 3D analog to the optical flow used in image data. A scene flow describes a change in motion of points between two consecutive point clouds. Formally, a scene flow is a vector field $F_{SF}\in R^{3\times N}$, which associates a motion $f_{i}\in F_{SF}$ with each point $p_{i}\in P_{final,t}$ from the point cloud $P_{final,t}=\left\{p_{1},p_{2},\ldots ,p_{N}\right\}$. It holds $p_{i}+f_{i}\approx p_{i}^{\prime }$, where $p_{i}^{\prime }\in P_{final,t+1}$ is a point of the subsequent point cloud [44]. A visualization of the scene flow is shown in Figure 11.

The vector field $F_{SF}$ can be formulated as an optimization problem as a sum over the N points of the point cloud [44]:

(35) $$\underset{F_{\mathrm{SF}}}{\min}\sum_{i=1}^{N}{\left\|\left(p_{i}+f_{i}\right)-p_{i}^{\prime }\right\|}^{2}.$$

Widely used methods for calculating a scene flow are learning-free algorithms based on the Iterative Closest Point (ICP) [45]. ICP tries to find corresponding points between two point clouds. However, due to the sparse sampling of the lidar scans or highly dynamic scene changes, as they occur in driving dynamics estimation, these directly corresponding points do not always exist. At this point, ICP methods reach their limits, while learning-based methods, which are able to map even complex motion patterns, offer clear advantages [46]. Our investigations based on a large number of environment recordings, including highly dynamic driving maneuvers, have confirmed the insufficient performance of ICP methods. Instead, we have decided to use the learning-based framework FLOT (Scene Flow on Point Clouds guided by Optimal Transport) [47], which is available as open source code. In FLOT, the deeper features are first extracted from two point clouds using CNNs, before the best matching correspondences are calculated based on optimal transport methods.

**Figure 12:**
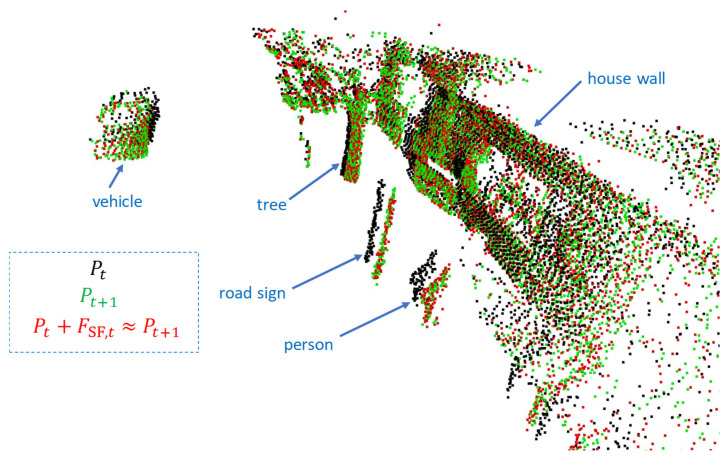
Visualization of a scene flow with oncoming traffic and various objects passing by. For plausibility checking, red dots (predictions) should match the green dots (ground truth).

The scene flow calculated with FLOT for two lidar frames recorded during one of our test drives is shown in Figure 12. The visualization also helps to check the plausibility: if the scene flow has been estimated correctly, $P_{t}+F_{SF,t}=P_{t+1}$ must apply. This means that the red dots shown in Figure 12 must match the green dots as closely as possible, which is the case. For the purpose of a better visualization of the scene flow, dynamic objects (vehicles and persons) were not filtered out.

### 2.4. State Estimation Using Recurrent Neural Networks

After all relevant quantities for estimating the desired vehicle states have been extracted from the perception sensors, they must be fused using an algorithm for state estimation. The advantages and drawbacks of learning- and model-based methods have already been discussed in Section 2.3.1.

Given the complexity of the perception sensor data and the extracted features (Section 2.1, Section 2.2 and Section 2.3) as well as the uncertainties and disturbances caused by the numerous necessary preprocessing steps, a learning-based method is used. For dynamic systems, recurrent neural networks (RNNs) have proven to be a powerful technique [48].

A nonlinear dynamic system is described by the temporal development of the state vector $x_{k}\in R^{n}$:

(36) $$\begin{array}{l} x_{k}=f_{k|k-1}\left(x_{k-1},u_{k-1}\right),\\ y_{k}=h\left(x_{k}\right),\\ x_{k}\in R^{n\times 1},u_{k}\in R^{s\times 1},y_{k}\in R^{m\times 1}. \end{array}$$

The goal of state estimation is to approximate the state vector ${\hat{x}}_{k}$ based on the observations $y_{k}$ as well as possible.

One of the most important features of RNNs is their ability to model temporal dependencies using a hidden state $s_{k}$, which acts as a memory and incorporates information from previous time steps. This allows an RNN to map the dynamics from Equation (36) with [49]

(37) $$\begin{array}{l} s_{k}=\phi \left(s_{k-1},y_{k}\right),\\ {\hat{x}}_{k}=\gamma \left(s_{k}\right), \end{array}$$

where the nonlinear functions $\phi \left(\cdot \right)$ and γ(⋅) are learned during the RNN training. An RNN can be considered a universal approximator for optimal filters such as the Kalman filter [49,50]. They offer a flexible data-based alternative for effective approximation of complex and nonlinear systems. It can be concluded that RNNs can be used not only in the context of state estimation, but also have a remarkable variety of applications and suitable architectures. Further information and detailed background can be found, for example, in [51].

## 3. Data Acquisition in Real-World Driving Tests

This chapter describes data collection with a test vehicle in real driving tests in order to train and analyze the framework presented in Section 2.

First, the test vehicle and the relevant hardware setup are presented, including the mounting of the perception sensors. Subsequently, the driving maneuvers performed are described, which represent the basis of the data acquisition and are used for both training and analysis of the estimator framework. Another focus of this chapter lies on the processing of the data recorded during the tests. Data synchronization plays a central role here, since the data is recorded at different sampling rates and processed on different systems. Finally, the minimum requirements for the state estimator’s sampling rate are discussed in the context of the considered vehicle dynamics problem.

### 3.1. Test Vehicle AI for Mobility and Its Hardware Setup

**Figure 13:**
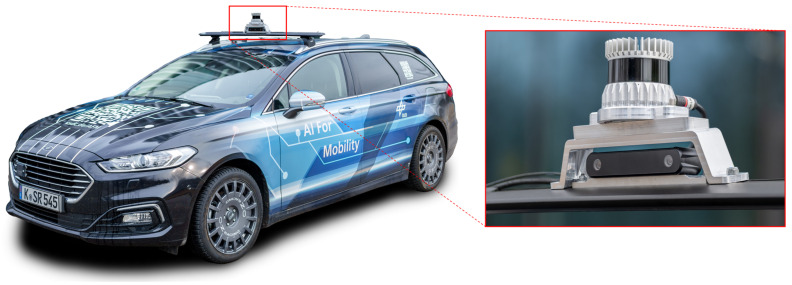
The test vehicle AI For Mobility (AFM) and its sensor carrier with Lidar and stereo camera.

The test vehicle AI For Mobility (AFM) is being designed by the Department of Vehicle System Dynamics and Control at the DLR to research AI-based vehicle control methods [42,52]. It is equipped with a complete drive-by-wire (DBW) system enabling the reproducible execution of automated driving maneuvers. To fulfill the requirements of data-driven methods for learning data, the vehicle is equipped with a variety of sensors. These include perception sensors as well as systems recording internal vehicle parameters and important vehicle dynamics quantities. The perception sensors relevant for this work include a stereo camera and a lidar sensor. However, it should be noted that the framework presented here is designed for broader applicability; therefore, only a monocular image of the stereo camera is used. The second image of the stereo camera remains unused. While the camera provides a field of view of the vehicle’s front area, the lidar enables coverage of the entire area around the vehicle thanks to its 360° detection range. Both sensors are installed on a sensor carrier on the front part of the vehicle roof (see Figure 13).

In addition to the perception sensors, precise measurement of the vehicle sideslip angle and vehicle velocity as a ground-truth measurement plays a central role. These measurements are provided by a high-precision navigation platform fusing inertial measurement unit (IMU) and GNSS data. Accuracy is further improved by considering real-time correction data via Real Time Kinematic (RTK). In addition, an optical system to measure the vehicle side-slip angle is used, which provides reliable data even in scenarios without GNSS availability. These sensors measure the vehicle sideslip angle $\beta^{c}$ and vehicle velocity $v^{c}$ with high accuracy. However, due to their high cost, they are typically used only in research vehicles such as the AFM, and not in series-production vehicles. Consequently, state estimators are required for practical applications. In this study, these sensors provide ground truth values for both training the RNN (supervised learning) and evaluating the performance of the vehicle state estimators.

The hardware setup of the test vehicle comprises two central systems: an embedded computer with a high-performance GPU (*NVIDIA Ampere GPU with 2048 CUDA and 64 Tensor cores, CPU: 12-core ARM Cortex-A78AE, RAM: 64 GB, up to 275 TOPS AI performance (NVIDIA (Santa Clara, CA, USA); ARM Ltd. (Cambridge, UK))*) and a rapid control prototyping (RCP) system. The embedded computer is used to record and process perception sensor data, while the vehicle controllers and the software for controlling the DBW system run on the RCP system. In addition, all vehicle-internal and driving dynamics quantities are logged in here. Both systems are connected to each other, enabling the synchronization of the sensor data (see Section 3.3.1). A detailed description of the hardware setup can be found in [42].

Regarding the preprocessing of sensor data, no action was taken except for the extraction of motion information from the perception sensors, specifically optical flow and depth information from camera images and scene flow from lidar point clouds (see Section 2.1, Section 2.2 and Section 2.3). To ensure data quality and consistency, the recorded data from the various sensors were subjected to random plausibility checks using simple transformations. Overall, the sensors provided reliable and consistent data.

### 3.2. Overview of the Driving Maneuvers

The test drives performed with the test vehicle AFM were mainly carried out on German country roads. During these test drives, data was recorded over a distance of more than 80 km and a duration of over 70 min. The focus of the test drives was on sections with relevant lateral dynamics, since the vehicle dynamics estimator was developed specifically for these areas. Particularly curvy sections, such as serpentine roads, provided ideal conditions for the application and validation of the estimator. To provide a challenging data basis, the maneuvers were performed to cover nonlinear driving dynamics regions. This included lateral accelerations of around $a_{y}^{c}\approx 8\;m/s^{2}$, well above the linear range of $a_{y}^{c}>4\;m/s^{2}$. Despite the focus on challenging driving scenarios, we made sure to create a representative data set. Therefore, fewer lateral-dynamic-relevant road sections, such as straight roads or long curves, were also included in the tests. This allowed us to cover all relevant areas of driving dynamics and ensure a comprehensive basis for the evaluation.

### 3.3. Data Synchronization and Analysis

As mentioned before, the sensor data is recorded on different systems and must be preprocessed before sensor fusion. The essential steps comprise time synchronization as well as analysis of the minimum required sampling times to operate the estimator in an error-free way in accordance with the Nyquist–Shannon sampling theorem.

#### 3.3.1. Data Synchronization

For the state estimator (RNN), all sensor data must be synchronized in time to fuse the input data (perception sensor data and yaw rate) with the output values (vehicle sideslip angle and velocity). This poses challenges due to the different sample rates: Lidar operates at 20 Hz (≙50 ms), the camera at 30 Hz (≙ 33.3 ms), and the RCP system at 1 kHz (≙1 ms). While a simple interpolation between measurements is applicable for continuous signals such as acceleration data, the synchronization of perception sensor data is more complex due to its structure. To ensure synchronous sensor signal processing, the sample rate of the state estimator framework is determined by the slowest sensor—in this case, the Lidar at 20 Hz. To synchronize to this rate, a method called “gating” is used. This involves opening a time gate each time a new point cloud is transmitted by the lidar sensor (every 50 ms). The nearest camera images and vehicle dynamics variables in time are then passed through this gate. Due to the high sampling rate of the RCP system (1 kHz), the logged vehicle dynamics parameters and lidar measurements are almost perfectly synchronized with a maximum error of 1 ms. In the worst case, the camera images are subject to a delay of up to one period (33.3 ms). However, this maximum delay is considered acceptable, since delays are usually shorter in practice. The method described is similar to the “Approximate Time Synchronizer” [53] as implemented in the ROS.

#### 3.3.2. Analysis of the Required Sampling Times

**Figure 14:**
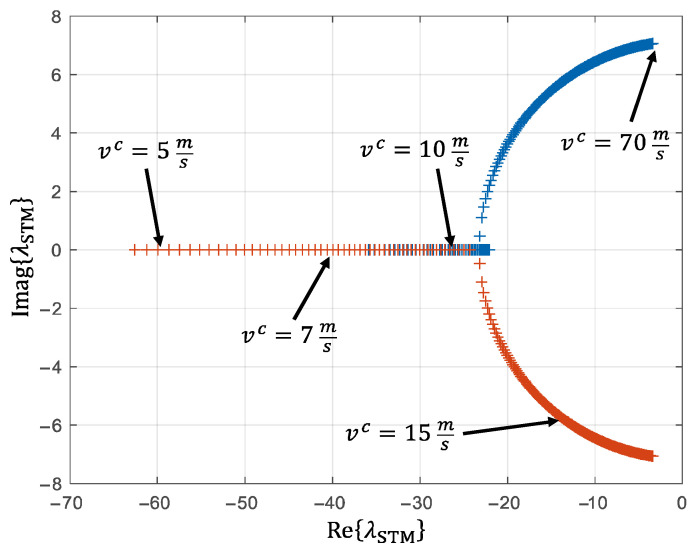
Vehicle velocity-dependent variation of the eigenvalues of the AFM single-track model.

After the time synchronization, the sensor data is available at a sampling rate of 20 Hz, corresponding to an interval of 50 ms. It is necessary to investigate whether the state estimator, in this case an RNN, can reliably estimate the vehicle sideslip angle and the vehicle velocity at this sampling rate without violating the Nyquist–Shannon sampling theorem. Therefore, the underlying dynamics must be examined. This corresponds to the lateral dynamics (vehicle sideslip angle) and the longitudinal dynamics (vehicle velocity). Since the vehicle lateral dynamics is considerably faster than its longitudinal dynamics, it is sufficient to consider the further estimation in relation to the lateral dynamics. A common model for describing vehicle lateral dynamics is the linear single-track model (STM). In state space representation, the vehicle sideslip angle and the vehicle yaw rate can be described as [54]

(38) $$\left[\begin{array}{c} \dot{\beta^{c}}\\ \ddot{\psi^{c}} \end{array}\right]=\underset{≔A}{\underbrace{\left[\begin{array}{cc} -\frac{c_{f}+c_{r}}{m\;v^{c}} & \frac{c_{r}\;l_{r}-c_{f}\;l_{f}}{m\;{v^{c}}^{2}}-1\\ \frac{c_{r}\;l_{r}-c_{f}\;l_{f}}{J_{z}} & \frac{c_{f}\;l_{f}^{2}+c_{r}\;l_{r}^{2}}{J_{z}\;v^{c}} \end{array}\right]}}\cdot \left[\begin{array}{c} \beta^{c}\\ \dot{\psi^{c}} \end{array}\right]+\left[\begin{array}{cc} \frac{c_{f}}{m\;v^{c}} & \frac{c_{r}}{m\;v^{c}}\\ \frac{c_{f}\;l_{f}}{J_{z}} & \frac{c_{r}\;l_{r}}{J_{z}} \end{array}\right]\cdot \left[\begin{array}{c} \delta_{f}\\ \delta_{r} \end{array}\right],$$

where $c_{f,r}$ represents the cornering stiffnesses at the front and rear axles, $l_{f,r}$ describes the distances to the CoG, $\delta_{f,r}$ denotes the steering angles at the front and rear wheel, and m and $J_{z}$ correspond to the vehicle mass and the yaw inertia, respectively. The eigenvalues $\lambda_{STM}$ of the dynamics matrix A parameterized for the AFM result in the values shown in Figure 14 as a function of the vehicle velocity.

The real part of the eigenvalues decreases with an increasing vehicle velocity, i.e., the system dynamics slow down. Via

(39) $$f_{STM}=\frac{\left|Re\left\{\lambda_{LSTM}\right\}\right|}{2\pi },$$

the real parts of the eigenvalues are related to the eigenfrequency of the respective system state. Based on this, the following estimation is made. For vehicle velocities $v^{c}>5\;\frac{m}{s}$, eigenvalues of $\left|Re\left\{\lambda_{LSTM}\right\}\right|<60$ result, which correspond to $f_{STM}<9.5$ Hz. The Nyquist–Shannon sampling theorem requires

(40) $$f_{Estimator}>2\cdot f_{System\;max},$$

where $f_{Estimator}=20$ Hz and $f_{System\;max}=f_{STM}=9.5$ Hz.

The Nyquist–Shannon sampling theorem is therefore also fulfilled for vehicle velocities $v^{c}>5\;\frac{m}{s}\;\left(18\;km/h\right)$ with the sensor signals downsampled to 20 Hz. This represents an acceptable limitation for the vehicle state estimator, since its scope of application lies in challenging driving maneuvers in the higher speed range.

## 4. Implementation and Evaluation

This chapter first presents the setup of the AI-based state estimator. This includes an overview of the RNN architecture, details regarding its structure and parameters, as well as information on its training. The AI-based estimator is compared to proven model-based state estimators, namely a Luenberger Observer and an Unscented Kalman Filter. Their setup is presented in Section 4.1.2. Next, we present a test scenario with a challenging and curvy mountain road that also includes a synthetic segment of an icy surface with a very low tire–road friction. The chapter concludes with the results achieved by the AI-based state estimator compared to those delivered by the model-based benchmark approaches.

### 4.1. State Estimator Setups

#### 4.1.1. AI-Based Vehicle State Estimator

The AI-based estimator is implemented using an RNN (see Section 2.4). In Section 2.3.1, the relative kinematic relationships between the ego vehicle and its environment are analyzed to determine the variables required from the camera and lidar sensors for estimating the vehicle sideslip angle and velocity. These variables are summarized in Table 2. The key inputs include motion information extracted from the camera images: optical flow in the longitudinal and lateral directions $F_{OF,x}$ and $F_{OF,y}$, respectively, along with the distance to flow objects $R_{MiDaS}^{c}$ and their associated longitudinal and lateral distances ${\Delta (x,y)}_{OF}^{c}=\left(\Delta x_{OF}^{c},\Delta y_{OF}^{c}\right)$. From the lidar sensor, inputs include the scene flow in all three spatial directions $F_{SF,x}$, $F_{SF,y}$ and $F_{SF,z}$, derived from lidar point clouds, as well as object distance $R_{Lidar}^{c}$ and the corresponding longitudinal and lateral distances ${\Delta (x,y)}_{SF}^{c}=\left(\Delta x_{SF}^{c},\Delta y_{SF}^{c}\right)$. Additionally, the vehicle yaw angle $\psi^{c}$ measured by the IMU (GNSS-aided) is used as an input variable.

**Figure 15:**
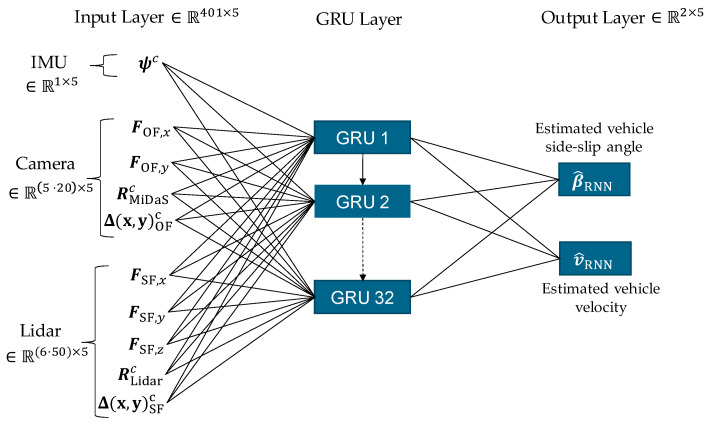
Overview of the architecture of the RNN, which is used as a state estimator for the vehicle side-slip angle and vehicle velocity based on IMU, camera, and lidar data.

As described in Section 2.3, not all object points from the camera and lidar need to be used as input variables due to significant redundancy. Instead, 20 image points are selected from the camera, each containing the extracted motion information, and 50 object points are chosen from the lidar sensor. With a sequence length of 5 time steps, this results in an input layer dimension of $R^{401\times 5}$. The RNN was trained using the Adam optimizer [55], which has proven effective for deep learning tasks. Gated Recurrent Units (GRUs) emerged as the optimal architecture for this application. An overview of the RNN architecture is provided in Figure 15.

The RNN was implemented in Python using the deep learning libraries Keras [56] and TensorFlow [57]. Training was conducted using the vehicle dynamics data described in Section 3. The dataset was partitioned into three disjoint subsets: training set $D_{train}$, validation set $D_{val}$, and test set $D_{test}$, with a distribution ratio of $\left|D_{train}\right|:\left|D_{val}\right|:\left|D_{test}\right|,=0.7:0.2:0.1$. Details regarding the test data are provided in Section 4.2.

To determine the best hyperparameters and network configuration, a comprehensive grid search was performed. The optimal parameters identified through this process are summarized in Table 3.

Additional parameters related to the architecture of the RNN network are presented in Table 4.

Thanks to the pre-computation of numerous variables from the perception sensor data, particularly the optical flow and scene flow, the network architecture is relatively lightweight. This design enables efficient and rapid training. On a high-performance personal computer (*CPU: Intel Core i9-13950HX, GPU: NVIDIA RTX3500 Ada, RAM: 64 GB, Keras/TensorFlow Version 2.11.x (Intel (Santa Clara, CA, USA); NVIDIA (Santa Clara, CA, USA); Samsung Electronics Co., Ltd. (Suwon, Republic of Korea))*), an average training session can be completed in under 60 min. The grid search optimization used to identify the optimal RNN architecture and parameterization was conducted on a dedicated in-house high-performance computing cluster.

Training was performed over a maximum of 500 epochs, employing early stopping mechanisms to prevent overfitting. Given the large size of the dataset, data generators were utilized during training to load data incrementally in batches, thereby reducing memory usage and ensuring efficient processing. Prior to the training, the input data was standardized using z-score normalization, scaling it to a mean of 0 and a standard deviation of 1.

#### 4.1.2. Model-Based Benchmark Estimators

To evaluate the performance of the AI-based vehicle state estimator, the results obtained are compared with those delivered by two model-based benchmark approaches.

**Unscented Kalman Filter based on a nonlinear two-track model**

The Unscented Kalman Filter (UKF) algorithm is widely regarded as one of the most advanced state estimation techniques for nonlinear systems within model-based approaches. In this work, a nonlinear two-track vehicle model parameterized for the AFM research vehicle serves as the prediction model. For tire modeling, a slightly simplified version of Pacejka’s Magic Formula 5.2 is used. The vehicle model is implemented in Modelica and integrated into an in-house-developed Kalman filter environment in MATLAB via the Functional Mock-up Interface (FMI) standard [58].

The UKF parameters are optimized within an optimization-based framework to ensure robust performance. Detailed descriptions of the vehicle model, Kalman filter environment, and parameterization methodologies are available in prior publications [59,60,61]. The UKF estimates three key states: the vehicle sideslip angle $\beta^{C}$, the vehicle velocity $v^{C}$ and the vehicle yaw rate ${\dot{\psi }}^{C}$ represented as:

(41) $$x_{UKF}={\left[\begin{array}{ccc} \beta^{C} & v^{C} & \begin{array}{c} {\dot{\psi }}^{C} \end{array} \end{array}\right]}^{T}.$$

The measurement vector includes the vehicle velocity $v^{C}$, longitudinal acceleration $a_{x}^{C}$, lateral acceleration $a_{y}^{C}$, and vehicle yaw rate ${\dot{\psi }}^{C}$ obtained from an inertial measurement unit (IMU):

(42) $$z_{UKF}={\left[\begin{array}{ccc} v^{C} & a_{x}^{C} & \begin{array}{c} \begin{array}{cc} a_{y}^{C} & {\dot{\psi }}^{C} \end{array} \end{array} \end{array}\right]}^{T}.$$

The input vector of the nonlinear two-track model comprises the wheel speeds $\omega_{Wheels}\in R^{4}$ and the front axle steering angles $\delta_{wheels}\in R^{2}$:

(43) $$u_{UKF}={\left[\begin{array}{cc} \omega_{Wheels} & \delta_{wheels} \end{array}\right]}^{T}.$$

In summary, this approach combines the well-established UKF algorithm for nonlinear systems with a high-fidelity nonlinear two-track model, resulting in a highly accurate yet computationally demanding vehicle state estimator.

**Luenberger Observer based on a linear single-track model**

In contrast to the previously described advanced approach utilizing a UKF combined with a nonlinear two-track model, a simpler Luenberger observer is employed based on a linear single-track model. This straightforward setup serves as a benchmark approach, requiring minimal parameterization effort and offering low computational complexity. It enables a comprehensive evaluation of the trade-off between model fidelity, computational effort, and estimation performance.

The linear single-track model used by the observer has been previously introduced in Section 3.3.2 (see state-space model in Equation (38)). This model estimates two key vehicle states—the vehicle sideslip angle $\beta^{C}$ and the yaw rate ${\dot{\psi }}^{c}$, represented as:

(44) $$x_{LB\;Obs}={\left[\begin{array}{cc} \beta^{C} & \begin{array}{c} {\dot{\psi }}^{C} \end{array} \end{array}\right]}^{T}.$$

The vehicle velocity $v^{c}$ is treated as a parameter within the time-variant system matrix A and is approximated as a quasi-input variable using the speed of the unsteered wheel rear left $\omega_{wheel,rl}$ and the wheel radius $R_{wheel}$:

(45) $${\hat{v}}_{LB\;Obs}^{c}=\omega_{wheel,rl}\cdot R_{wheel}.$$

The vehicle yaw rate ${\dot{\psi }}^{c}$ is utilized as the observation variable, resulting in an initial output vector $c={\left[\begin{array}{cc} 0 & 1 \end{array}\right]}^{T}$. The observer gain was determined through the pole placement method, ensuring stable and reliable estimation performance.

#### 4.1.3. Test Scenario

To evaluate the performance of the AI-based vehicle state estimation and compare it with model-based approaches, a test track, which the recurrent neural network (RNN) had not previously encountered, was selected. The test dataset comprises approximately 10% of the total database, and is entirely independent of the training and validation datasets (see Section 3.2 and Section 4.1.1). This ensures that the results reflect the model’s performance on an unseen route.

The test track is located in the German Alpine foothills and includes a mountain road featuring diverse road types. It consists of hairpin turns, winding sections, long curves, and isolated straight segments. The vehicle speeds $v^{c}$ range from 8 m/s to 22 m/s, while the lateral acceleration magnitude $\left|a_{y}^{c}\right|$ reaches up to 8 m/s^2^. Consequently, the test scenario includes highly nonlinear regions where $\left|a_{y}^{c}\right|>4\;m/s^{2}$. The entire test drive lasts approximately 6 min. Two specific sections were selected for detailed analysis as described below.

In test section Ⓐ, the performance of the state estimators is analyzed under ideal road conditions. This segment features a dry road with high grip and a friction coefficient of approximately $\mu_{tire\;road}\;\approx \;1$.

Test section Ⓑ examines a critical driving scenario. To simulate low tire–road friction conditions $\mu_{tire\;road}\;\approx \;0$, some measurement data was synthetically modified to represent a “zero-grip” surface where no traction exists between the tires and the road. Such conditions would be extremely hazardous in real-world driving scenarios. In this case, conventional signals like steering wheel angles and wheel speeds lose their reliability as indicators of actual vehicle motion. For this synthetic low-friction representation, affected measurements were adjusted accordingly. To mimic the behavior of ESC under these conditions, simplified interventions were modeled: wheel speeds were held constant (representing reduced drive power), and steering angles were fixed at 0°. This simplification isolates vehicle behavior in critical scenarios without introducing complex vehicle dynamics control mechanisms.

**Figure 16:**
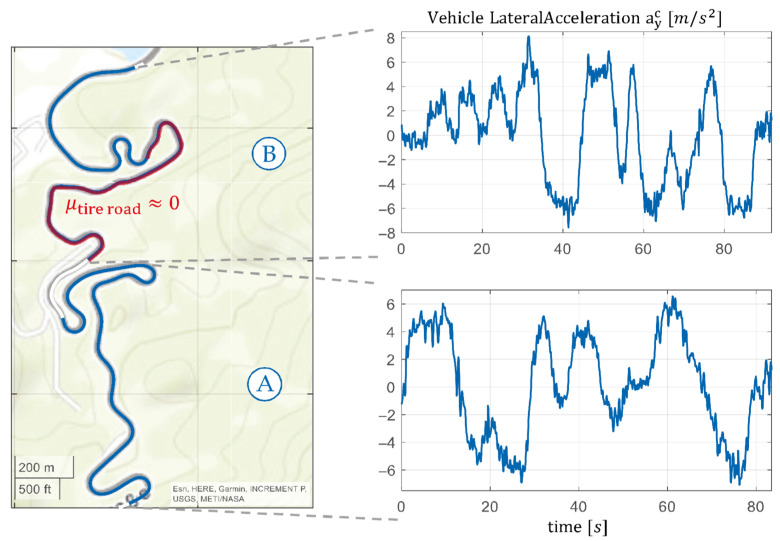
Visualization of the test scenario: the selected sections of the driven route are shown on a road map (**left**), alongside the corresponding vehicle lateral accelerations (**right**). Test section Ⓐ features ideal road conditions, while section Ⓑ represents with a critical area with low tire-grip.

Figure 16 provides an overview of the driven test track and highlights the two analyzed sections, Ⓐ and Ⓑ. Additionally, the corresponding lateral accelerations of the vehicle are shown to illustrate the dynamic characteristics of each section.

This test scenario enables a comprehensive evaluation of the vehicle state estimation’s performance under both normal and extreme driving conditions, providing insights into its robustness and accuracy.

### 4.2. Results

This section presents the estimation results of the vehicle sideslip angle $\beta^{c}$ and vehicle velocity $v^{c}$ in the test scenario described in Section 4.2. AFM, which was previously introduced in Section 3.1, serves as the test vehicle.

For the AI-based approach, the principal computational burden lies in the preprocessing of sensor data. In contrast, the RNN component is notably efficient, owing to its architecturally optimized design based on a rigorous analysis of the underlying system dynamics (see Section 2.3.1). It should be emphasized that model-based approaches achieve real-time execution at 20 Hz on the AFM’s embedded computing system. Although full real-time inference with the AI-based method has not yet been validated in this study, the integration of GPU hardware in the test vehicle suggests that real-time performance is feasible. This potential will be explored in future investigations.

The AI-based approach that utilizes an RNN is compared with the two model-based benchmark methods: a UKF and a Luenberger observer. The results are first analyzed for test section Ⓐ, characterized by a high tire–road friction coefficient, followed by the more challenging test section Ⓑ, which features a low tire–road friction coefficient.

To assess the estimation quality, two error metrics are employed: the root-mean-squared error (RMSE), which provides a physically interpretable measure of error magnitude, and the fit value [62], which quantifies the percentage agreement between the estimated and reference signals. A fit value of 100% represents a perfect match, offering a straightforward and intuitive interpretation of the estimation accuracy.

#### 4.2.1. High Tire–Road Friction

**Figure 17:**
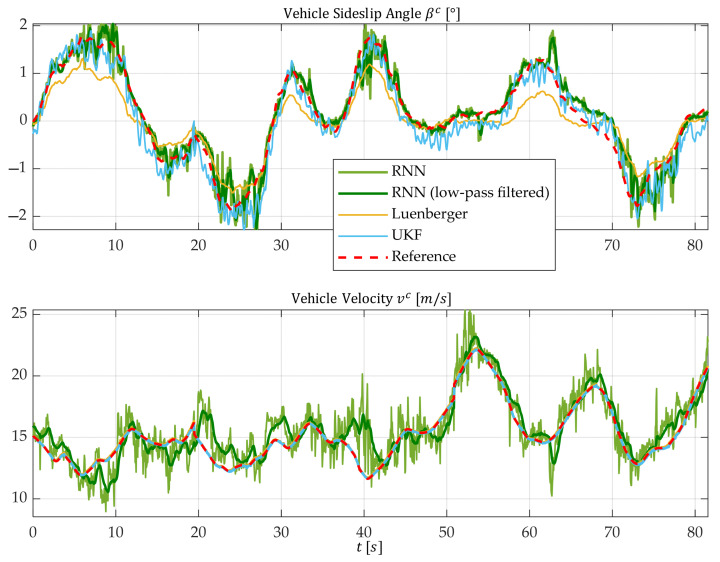
Time profile of the actual vehicle states (reference) as well as the profiles of three vehicle state estimators for test section Ⓐ with a high friction value.

Figure 17 illustrates the time profiles of the vehicle sideslip angle $\beta^{c}$ and vehicle velocity $v^{c}$ for test section Ⓐ characterized by a high friction coefficient between the tires and the road surface.

The associated error values for the three approaches are shown in Table 5.

The Luenberger observer, which relies on a linear single-track model and operates with a minimal sensor setup, significantly underestimates the vehicle sideslip angle $\beta^{c}$. This behavior is expected, as the driving conditions fall within the nonlinear vehicle dynamics range. The linear single-track model assumes a proportional relationship between tire lateral forces and tire slip angles. However, this assumption becomes invalid at lateral accelerations of $\left|a_{y}^{c}\right|>4\;m/s^{2}$.

In contrast, the UKF provides highly accurate estimates of the vehicle sideslip angle amplitudes. This accuracy stems from the UKF’s foundation on a nonlinear two-track model, which enables it to capture complex tire saturation effects at the limits of driving dynamics.

The AI-based state estimation approach achieves similar or even slightly better results compared to the UKF. The advantages of the AI approach are particularly pronounced in highly dynamic driving scenarios.

For the estimation of the vehicle velocity $v^{c}$, both model-based methods (UKF and Luenberger observer), which use tire speeds as input variables (see Equation (45)), demonstrate exceptional precision. In comparison, the AI-based approach exhibits relatively noisy results with sporadic offsets. However, these high-frequency disturbances can be effectively mitigated by applying a low-pass filter without introducing noticeable time delays. It can be observed that the AI-based approach yields higher estimation accuracy for the vehicle sideslip angle compared to vehicle velocity. Notably, in the interval from just before t=10 s to just before t=40 s (see the lower plot in Figure 17), the model predicts a decreasing or increasing vehicle velocity, whereas the actual value exhibits the inverse trend. Due to the black-box nature of the RNN, a precise explanation for this behavior remains undetermined. Nevertheless, it is important to note that such discrepancies manifest only sporadically within the operational range.

#### 4.2.2. Low Tire–Road Friction

Test section Ⓑ includes a segment with a low friction coefficient $\mu_{tire\;road}\approx 0$, where no traction exists between the tires and the road surface. Under such conditions, the tire sensor data can no longer provide reliable information for motion estimation. This scenario represents a significant challenge for vehicle state estimation, as accurate state predictions are critical for stabilizing interventions by vehicle dynamics controllers in potentially hazardous situations.

**Figure 18:**
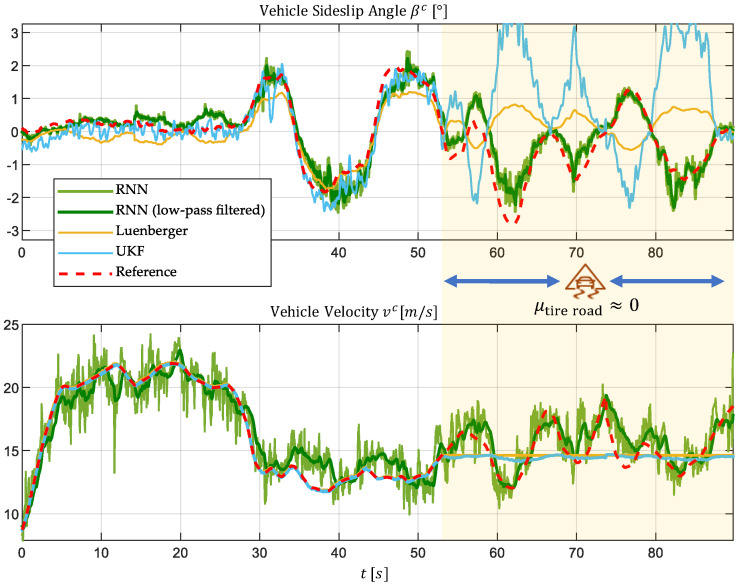
Time profiles of the true vehicle states (reference) and the estimated states from the three vehicle state estimation approaches for test section Ⓑ, including a subsection with a low friction coefficient where traction between tires and the road is lost.

Figure 18 illustrates the time profiles of the true vehicle states (reference) alongside the estimates produced by the three approaches for this test section.

The corresponding error values for this test section are summarized in Table 6.

The two model-based approaches (UKF and Luenberger observer) rely on input variables such as tire speeds and wheel steering angles. In the area without traction, these inputs become worthless, resulting in implausible estimates, as expected. Although complete divergence is prevented by supplementary data from the IMU, both the magnitudes and signs of the estimated vehicle sideslip angle $\beta^{c}$ are incorrect.

For vehicle velocity $v^{c}$, the model-based approaches maintain a constant estimate at the last known value. This behavior arises because the intervention of the synthetically generated vehicle dynamics controller holds wheel speeds constant in this scenario (see Section 4.2). Consequently, reasonable velocity estimates are not achievable under these conditions.

In contrast, the AI-based approach using an RNN demonstrates robustness in this challenging scenario. By leveraging perception sensors, it operates independently of the unreliable wheel sensor data and continues to provide plausible estimates for both the vehicle sideslip angle and velocity. Although the velocity estimates exhibit relatively high-frequency noise, it can be effectively reduced through low-pass filtering without significant time delays.

Note that the estimation accuracy of the vehicle sideslip angle is lower in this case compared to the previous section, where a high friction coefficient was assumed. This effect is incorporated in the error values presented in Table 6. It should, however, be emphasized that this decrease in accuracy does not stem from the change in the tire–road friction and is rather related to the challenging conditions in this section. Here, both lateral and longitudinal dynamics undergo rapid changes, as evidenced by the oscillatory behavior of the vehicle sideslip angle and vehicle velocity shown in Figure 18.

#### 4.2.3. Results Overview

The AI-based vehicle state estimator utilizing a recurrent neural network (RNN) and perception sensors delivers comparable or even slightly superior estimates of the vehicle sideslip angle in high-friction conditions compared to the high-performance benchmark approach—UKF paired with a nonlinear two-track model and conventional sensors. Furthermore, the AI estimator performs exceptionally well in nonlinear driving dynamics regions, where the linear Luenberger observer significantly underestimates the vehicle sideslip angle.

In low-friction conditions, where the tires lose their traction potential, the model-based approaches fail entirely. They are unable to produce plausible estimates for either the vehicle sideslip angle or vehicle velocity. In contrast, the AI-based estimator remains robust under these challenging circumstances. By leveraging perception sensors, it bypasses reliance on the dynamic system of tires and their sensor data, enabling reliable state estimation across diverse scenarios.

A drawback of the AI estimator becomes apparent in vehicle velocity estimation. The results exhibit noise components, but these can be effectively mitigated by applying a low-pass filter without introducing significant delays.

In summary, the AI-based approach, which leverages environmental sensor data, demonstrates clear advantages over the two model-based methods, particularly in critical scenarios involving low tire–road friction coefficients. Its robustness and independence from tire sensor data make it a promising solution for reliable vehicle state estimation under extreme driving conditions.

## 5. Summary and Outlook

This work introduces a powerful AI-based framework for vehicle state estimation that utilizes perception sensors and the vehicle yaw angle. The estimator primarily builds upon the motion-related data extracted from perception sensors, including the optical flow from 2D camera images and the scene flow from 3D point clouds.

To enhance transparency and optimize the network architecture, the mathematical relationship between the perception sensor data and vehicle states was derived. The state estimator is based on a recurrent neural network (RNN) and benchmarked against two model-based approaches: the Unscented Kalman Filter (UKF), which utilizes a nonlinear two-track model, and a linear Luenberger observer.

A major advantage of the AI-based estimator lies in its utilization of perception sensors, enabling it to bypass the complex vehicle and tire model, which is a primary source of nonlinearities in conventional model-based vehicle state estimation. Tire models are highly sensitive to external factors such as temperature, tire wear, and road conditions, introducing significant uncertainties into traditional estimation methods. By leveraging perception sensors and utilizing relative kinematic relationships, the AI approach overcomes this source of uncertainty.

The results demonstrate that the AI estimator provides robust and reliable estimates. Particularly in scenarios with very low friction coefficients between tires and the road surface, such as icy driving conditions, its advantages over model-based approaches become evident. In these scenarios, model-based approaches fail due to their dependence on wheel sensor data and become, therefore, unusable. Additionally, the AI-based approach offers another significant advantage: its independence from vehicle-specific parameters. Once trained, the model can be seamlessly transferred to other vehicles without requiring extensive recalibration.

Future work will focus on integrating additional perception sensors, such as radar, into the estimation process to further enhance the robustness and accuracy. Moreover, test scenarios will be extended to include challenging environmental conditions such as darkness, rain, or snow to evaluate performance under real-world adverse conditions. Additionally, the dataset will be extended to include other challenging driving maneuvers, such as step-steer inputs and emergency braking, enabling a more comprehensive evaluation of system robustness.

Full real-time implementation of the entire AI-based estimator toolchain on the vehicle’s onboard computing systems is planned. In this context, particular attention will be given to assessing the impact of computationally intensive preprocessing steps on the overall system performance.

To further enhance the estimation quality, the AI-based approach will be integrated with model-based methods to develop a hybrid estimator that harnesses the respective benefits of both strategies.

## Figures and Tables

**Table 2:** Overview of the quantities required by the perception sensors to estimate the vehicle sideslip angle $\beta^{c}$ and the vehicle velocity $v^{c}$ in order to meet the underlying relative kinematic relationships.

Description	Variable	Camera Image (Mono)	Lidar Point Cloud
Distance between vehicle and object	$R^{c}$	Must be determined by depth estimation	Measured directly
Longitudinal and lateral distance vehicle to object	$\Delta x^{g},\Delta y^{g}$	Determined using the object’s pixel coordinates and the estimated depth (see Section 2.3.2)	Measured directly
Change of position of object relative to vehicle	$\dot{\Delta x^{g}},\dot{\Delta y^{g}}$	Related to the optical flow of an object point	Scene flow—the 3D analog of the optical flow

**Table 3:** Summary of the optimal hyperparameters of the RNN determined by the grid search procedure.

Parameter	RNN Type	Hidden Layers	Sequence Length	GRU Units	Dropout Rate		Learning Rate
					Feedforward	Recurrent	
**Value**	GRU	1	5	32	0.5	0.3	0.001

**Table 4:** Summary of the non-optimized parameters of the RNN network architecture and information about the training.

Parameter	Activation Function			Loss Function	Batch Size	Volume of Training Data	
	State	Gate	Output			Time	Distance
**Value**	tanh	sigmoid	linear	Mean Squared Error	60	65 min	80 km

**Table 5:** Error metrics for each vehicle state estimation approach for test section Ⓐ with a high tire–road friction.

Criteria	Fit [%]		RMSE	
State	${\hat{\beta }}^{C}$	${\hat{v}}^{C}$	${\hat{\beta }}^{C}$ [°]	${\hat{v}}^{C}$ [m/s]
**Estimator**				
RNN (low-pass filtered)	73.7	49.7	0.25	1.19
Unscented Kalman Filter	70.5	97.4	0.28	0.06
Luenberger Observer	58.6	97.1	0.38	0.06

**Table 6:** Error metrics for the three vehicle state estimation approaches in test section Ⓑ, subdivided into segments with a high and a low tire–road friction.

Section	High Tire Road Friction				Low Tire Road Friction			
Criteria	Fit [%]		RMSE		Fit [%]		RMSE	
State	${\hat{\beta }}^{C}$	${\hat{v}}^{C}$	${\hat{\beta }}^{C}$ [°]	${\hat{v}}^{C}$ [m/s]	${\hat{\beta }}^{C}$	${\hat{v}}^{C}$	${\hat{\beta }}^{C}$ [°]	${\hat{v}}^{C}$ [m/s]
**Estimator**								
RNN (low-pass filtered)	65.3	71.7	0.33	1.1	49.2	30.1	0.46	1.1
Unscented Kalman Filter	64.4	98.4	0.33	0.1	−200	−56.6	2.92	1.7
Luenberger Observer	55.8	98.3	0.41	0.1	−66.3	−53.2	1.52	1.7

## Data Availability

The original contributions presented in the study are included in the article. Further inquiries can be directed to the corresponding author.

## Appendix A. Supplementary Derivations of the Relative Kinematics Equations

**Geometric Derivation of the Look Angle Rate**

This section provides supplementary derivations to Equations (20)–(24) in Section 2.3.1, based on the geometric relationships illustrated in Figure 8.

The component of the vehicle’s velocity perpendicular to the line of sight (LOS) is given by

(A1) $$v_{⊥LOS}^{c}=v^{c}\sin\left(\lambda_{z}^{c}-\beta^{c}\right).$$

The component of the vehicle’s velocity parallel to the line of sight is:

(A2) $$v_{∥LOS}^{c}=v^{c}\cos\left(\lambda_{z}^{c}-\beta^{c}\right).$$

The line of sight angle $\sigma^{c}$ can be expressed in terms of the vehicle yaw angle $\psi^{c}$ and the look angle $\lambda_{z}^{c}$ as follows:

(A3) $$\sigma^{c}=\psi^{c}+\lambda_{z}^{c}.$$

The line of sight rotation rate is determined by the perpendicular velocity component $v_{⊥\;LOS}^{c}$ and the distance to the object $R^{c}$:

(A4) $${\dot{\sigma }}^{c}=\frac{v_{⊥LOS}^{c}}{R^{c}}.$$

By differentiating Equation (A3) with respect to time and rearranging for the look angle rate, we obtain:

(A5) $${\dot{\lambda }}_{z}^{c}={\dot{\sigma }}^{c}-{\dot{\psi }}^{c}.$$

Substituting Equation (A4) into Equation (A5), the geometrically derived expression for the look angle rate is:

(A6) $${\dot{\lambda }}_{z}^{c}=\frac{v^{c}}{R^{c}}\sin\left(\lambda_{z}^{c}-\beta^{c}\right)-\dot{\psi }.$$

**Approach Velocity**

The approach velocity is defined as the negative rate of change of the distance to the object $R^{c}$ and can be expressed in terms of the velocity component parallel to the line of sight (see Equation (A2)):

(A7) $${\dot{R}}^{c}=-v_{∥LOS}^{c}=-v^{c}\cos\left(\lambda_{z}^{c}-\beta^{c}\right).$$

## Appendix B. Technical Data of the Perception Sensors

**Property**	**Lidar Sensor**	**Camera**
Effective Range	90 m	20 m
Vertical Field of View	45 °	54°
Horizontal Field of View	360°	84°
Resolution	65,536 points per scan (64 vertical channels, 1024 points per channel)	1920×1080 Pixel
Frame Rate	20 Hz	30 Hz
Hardware Interface	Ethernet	USB Type-C

## Appendix C. Lists of Symbols, Nomenclature, and Abbreviations

**Formula Symbol**	**Description**
$a_{x,y}^{c}$	Vehicle’s acceleration (longitudinal or lateral)
$\beta^{c}$	Vehicle’s side slip angle
$\delta_{wheel}$	Wheel steering angle
${\Delta (x,y)}_{OF}^{c}$	Distance between Vehicle and Optical Flow Point (Longitudinal and Lateral)
$\Delta x^{g},\Delta y^{g}$	Distance between Vehicle and Object (Longitudinal and Lateral)
$F_{OF}$	Optical flow vector (2D)
$F_{SF}$	Scene flow vector (3D)
I	Image matrix (Pixels)
$\lambda_{z}^{c}$	Look Angle between the Horizontal Line and the Line of Sight to an Object
M	Image Mask (Binary Matrix)
$\mu_{tire\;road}$	Coefficient of friction between tire and road
O	Cluster of Points from Point Cloud
$\omega_{wheel}$	Wheel speed
p	Individual Point from Pointcloud (3D Vector)
P	Point cloud (3D)
$\psi^{c}$	Vehicle’s yaw angle
R	Distance between Vehicle and Object Point
$v^{c}$	Vehicle’s velocity over ground

**Abbreviation**	**Explanation**
ADAS	Advanced Driver Assistance Systems
AFM	AI For Mobility (DLR research vehicle)
CNN	Convolutional neural network
CoG	Center of gravity
DLR	German Aerospace Center
ESC	Electronic Stability Control
GNSS	Global Navigation Satellite System
GRU	Gated Recurrent Unit
ICP	Iterative Closest Point
IMU	Inertial measurement unit
LOS	Line of sight
MiDaS	Monocular Depth Estimation via Scale
RNN	Recurrent neural network
ROI	Region of interest
ROS	Robot Operating System
STM	Single-track model
UKF	Unscented Kalman Filter
VDC	Vehicle dynamics control
YOLO	You Only Look Once

**Nomenclature**	**Explanation**
${\left(\cdot \right)}^{c}$	Quantity expressed in the car coordinate system with origin in CoG
${\left(\cdot \right)}^{g}$	Quantity expressed in the geodetic coordinate system
${\left(\cdot \right)}^{img}$	Quantity expressed in the image coordinate system (pixels)
$\hat{\left(\cdot \right)}$	Estimated state
